# Vaccinia-related kinase 2 confers pancreatic cancer with gemcitabine resistance through TPI1-driven aerobic glycolysis

**DOI:** 10.1038/s41420-026-03303-8

**Published:** 2026-08-17

**Authors:** Hengqing Zhu, Bin Xu, Ruiying Zhu, Jiajuan Li, Qing Li, Yucheng Qian, Zhiyu Li, Sisi Chen

**Affiliations:** 1 Department of Thyroid Surgery, Second Affiliated Hospital School of Medicine, Zhejiang University, Hangzhou, China; 2 Center for Medical Research and Innovation in Digestive System Tumors, Minstry of Education, Hangzhou, China; 3 Department of Burns, First Affiliated Hospital of Nanchang University, Nanchang, China; 4 College of Medicine, Zhejiang University, Hangzhou, China; 5 Department of Infectious Disease, Second Affiliated Hospital of Nanchang University, Nanchang, China; 6 Department of Pathology, Second Affiliated Hospital of Nanchang University, Nanchang, China; 7 Department of Neurology, Second Affiliated Hospital of Nanchang University, Nanchang, China

**Keywords:** Cancer therapy, Cell death

## Abstract

Gemcitabine(GEM)-based chemotherapy is considered the first-line regimen for pancreatic cancer(PC), but the poor responsiveness and chemoresistance greatly limit its application. Metabolic reprogramming, especially aerobic glycolysis, which is regulated by glycolytic enzymes, is closely associated with the malignant behavior of PC. Recently, triosephosphate isomerase 1(TPI1), as a key glycolytic enzyme involved in glycolysis, was reported to be increased in GEM-treated PC patients with poor outcome whereas the mechanism remains unclear. In this study, we identified vaccinia-related kinase 2 (VRK2), a serine/threonine protein kinase, which was upregulated in GEM-resistant PC cells and conferred GEM resistance by promoting TPI1-driven aerobic glycolysis. Further studies showed that phosphorylation and nuclear translocation of the transcriptional factor SP1 induced by VRK2 contributed to the interaction of SP1 with the promoter region of TPI1 and thus enhanced the transcriptional activity of TPI1. Taken together, our findings uncover a novel role for VRK2 in modulating glycolytic reprogramming and GEM-resistance, which implicates VRK2 as a potential therapeutic target in PC.

**Figure:**
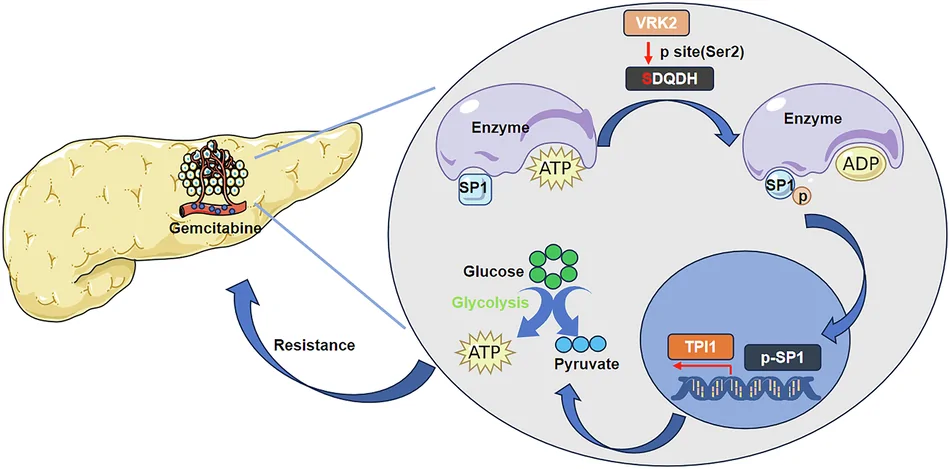

## Introduction

Pancreatic cancer (PC), characterized by its aggressive malignancy and limited treatment options, represents a significant global health challenge [1, 2]. Chemotherapy serves as a cornerstone approach for patients with locally advanced or metastatic disease while also functioning as a neoadjuvant strategy to enhance surgical resectability [3]. Gemcitabine (GEM)-based regimens currently constitute first-line therapy for improving PC patient outcomes [4]. However, inherent chemoresistance and poor treatment response continue to pose substantial clinical barriers to effective chemotherapy implementation [5]. Elucidating the molecular mechanisms underlying chemoresistance may therefore offer novel insights for developing alternative therapeutic strategies.

Aerobic glycolysis, also known as the Warburg effect, represents a metabolic hallmark of cancer characterized by the preferential utilization of glycolysis for energy production even under normoxic conditions [6]. This aberrant metabolic reprogramming, primarily driven by dysregulated glycolytic enzyme expression, not only fuels tumor proliferation but also confers malignant phenotypes such as chemoresistance [7, 8]. Among these metabolic regulators, triosephosphate isomerase 1 (TPI1) serves as a critical rate-limiting enzyme in glycolysis, efficiently catalyzing the reversible interconversion between glyceraldehyde-3-phosphate (GAP) and dihydroxyacetone phosphate (DHAP), thereby redirecting metabolic flux toward glycolysis to meet tumor cells’ heightened energetic demands [9]. The protein homeostasis and enzymatic activity of TPI1 are tightly regulated within the tumor microenvironment. Mechanistic studies reveal that the deubiquitinase USP5 specifically recognizes and removes ubiquitin modifications from TPI1, significantly prolonging its protein half-life [10]. LKB1 kinase-mediated phosphorylation of TPI1 has been identified as a molecular switch for activating its enzymatic function [11]. Additionally, emerging evidence reveals that TPI1 exhibits context-dependent functions in tumorigenesis [9, 12]. For example, TPI1 acts as a tumor suppressor when downregulated in hepatocellular carcinoma, whereas its overexpression and nuclear location in lung adenocarcinoma promotes oncogenic behaviors including proliferation, migration and chemoresistance [13, 14]. Notably, emerging evidence suggests TPI1 plays a role in PC progression, particularly as recent clinical observations demonstrate elevated serum TPI1 levels correlate with unfavorable prognosis in GEM-treated PC patients [15]. However, the underlying regulatory mechanisms and its precise biological significance remain incompletely elucidated.

Protein phosphorylation, as a pivotal post-translational modification, serves as a molecular switch orchestrating diverse biological processes through the catalytic action of protein kinases. Among these, the Vaccinia-related kinase (VRK) subfamily of serine-threonine kinases has garnered significant attention due to its distinctive expression patterns in human brain tissues and highly proliferative cell compartments, including testes, thymus, and fetal hepatocytes [16, 17]. VRK2, a critical member of this family, exhibits oncogenic properties with well-documented roles in tumor progression. For instance, it enhances breast cancer cell invasiveness via NFAT1 phosphorylation and confers sorafenib resistance in hepatocellular carcinoma by disrupting the apoptosis-autophagy equilibrium [18, 19]. Notably, Current and our previous studies have identified VRK2 upregulation in PC as a key driver of tumorigenesis [20, 21]. However, its involvement in chemoresistance—particularly to GEM—remains unexplored. Herein, our study reveals that ‌VRK2 overexpression is a hallmark of GEM-resistant PC cells‌, directly contributing to chemoresistance. Mechanistically, VRK2 promotes aerobic glycolysis by transcriptionally upregulating TPI1, thereby fueling metabolic reprogramming essential for GEM resistance. These findings not only elucidate VRK2’s novel regulatory role in glycolytic metabolism but also position it as a promising therapeutic target for overcoming PC chemoresistance.

## Results

### Elevated expression of VRK2 in GEM-resistant PC cells and clinical samples

**Fig. 1:**
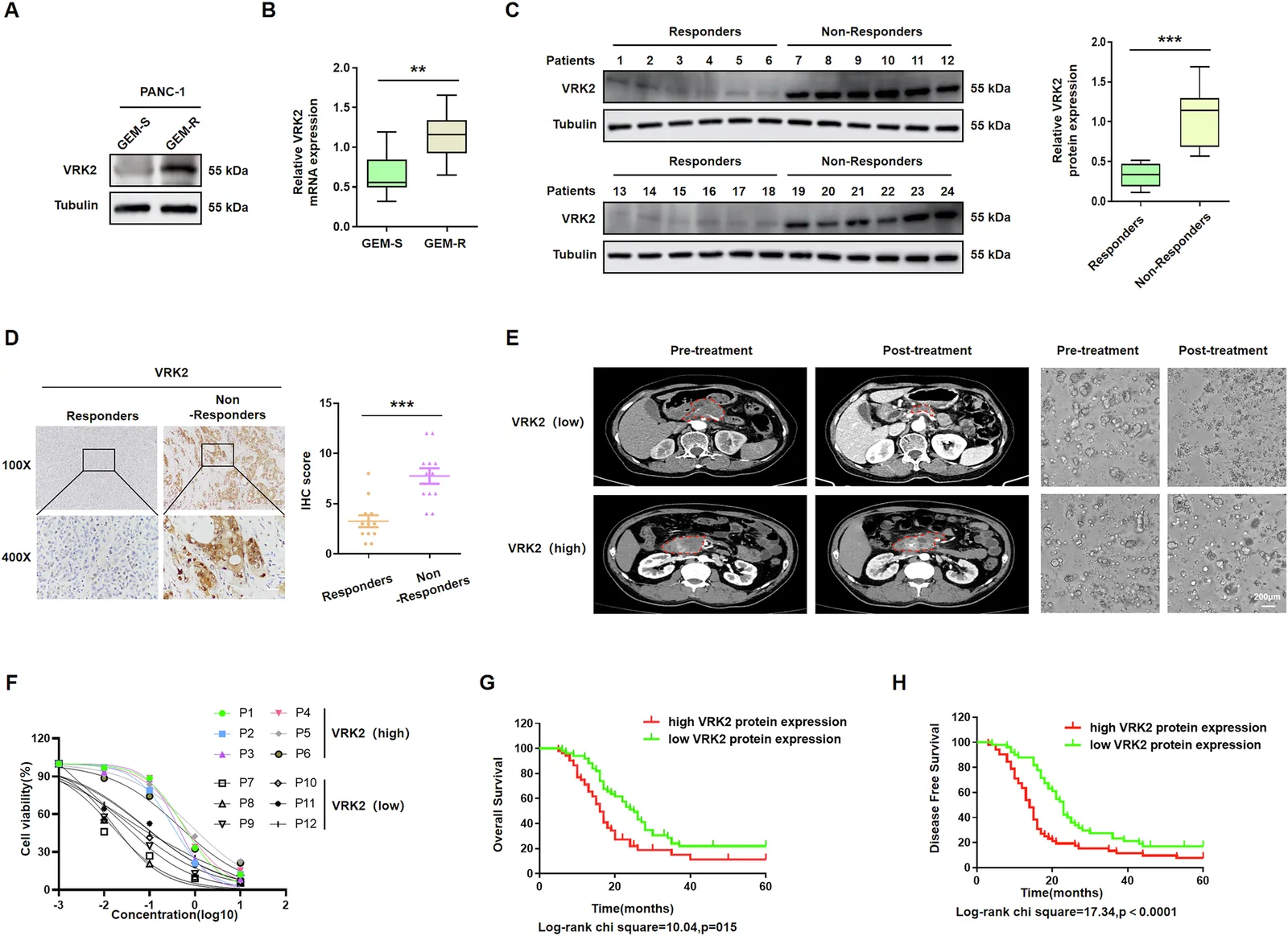
Elevated expression of VRK2 in GEM-resistant PC cells and clinical samples. **A** Immunoblot for protein expression of VRK2 in GEM-sensitive and GEM-resistant PANC-1 cells during the treatment with GEM (GEM-resistant PANC-1, 5 μΜ; GEM-sensitive PANC-1, 2 μΜ). **B** mRNA of VRK2 in GEM-sensitive and GEM-resistant PANC-1 cells during the treatment with GEM (GEM-resistant PANC-1, 5 μΜ; GEM-sensitive PANC-1, 2 μΜ) tested by RT-qPCR. *n* = 3; **p < 0.01. **C** Immunoblot for protein expression of VRK2 in PC tissues from GEM responders and GEM non-responders. ***p < 0.001. **D** IHC analysis of VRK2 expression in PC tissues from GEM responders and GEM non-responders. ***p < 0.001. For 100×figures, the scale bar indicates 100 μm; For 400×figures, the scale bar indicates 50 μm. **E** Representative pancreas CT and organoid images of responder and non-responder treated by GEM. The red dashed circle indicates a CT scan of the tumor lesion in the pancreas in PC patients. **F** The IC50 values of PC patient-derived organoids treated with GEM determined by CCK8 assay (*n* = 6 for VRK2-high and *n* = 6 for VRK2-low expression groups): 0.5404 (0.3276-0.8603) VS 0.04363 (0.01515-0.07998) *p* <0.001. **G, H** Kaplan–Meier analysis of overall survival curves and disease-free survival curves of PC patients with varying VRK2 expression levels who received GEM treatment.

To explore the role of VRK2 in GEM-resistant PC, we first established two GEM-resistant PC cell lines (Supplementary Fig. 1A, B) and examined the expression of VRK2 in GEM-resistant PC cells and GEM-sensitive PC cells using western blot and qRT-PCR. We found that VRK2 was significantly upregulated in GEM-resistant PC cells when compared to GEM-sensitive PC cells (Fig. 1A, B and Supplementary Fig. 1C, D). To validate these findings in PC tissues, we analyzed VRK2 expression in a cohort of 12 GEM responders and 12 non-responders. Patients were evaluated bimonthly by computed tomography (CT) using modified RECIST criteria to assess treatment response. Western blot analysis revealed significantly elevated VRK2 expression more frequently in tissues from GEM-resistant patients (Fig. 1C). Immunohistochemical (IHC) staining showed higher average VRK2 staining scores in tissues from GEM-insensitive patients compared to responders (Fig. 1D). Consistent with these observations, CT assessment indicated that PC patients with high VRK2 expression exhibited GEM resistance. Furthermore, compared to organoids derived from VRK2-low-expressing tissues, those from VRK2-high-expressing PC tissues exhibited significantly reduced sensitivity to GEM, accompanied by a substantial increase in GEM IC50 (Fig. 1E, F). In this small clinical cohort, Kaplan–Meier analysis revealed exploratory associations between VRK2 expression and overall and disease-free survival in patients undergoing GEM treatment (Fig. 1G, H).These preliminary data suggest that VRK2 may act as a potential indicator for GEM response in PC.

### VRK2 promotes the GEM resistance in vitro and in vivo

**Fig. 2:**
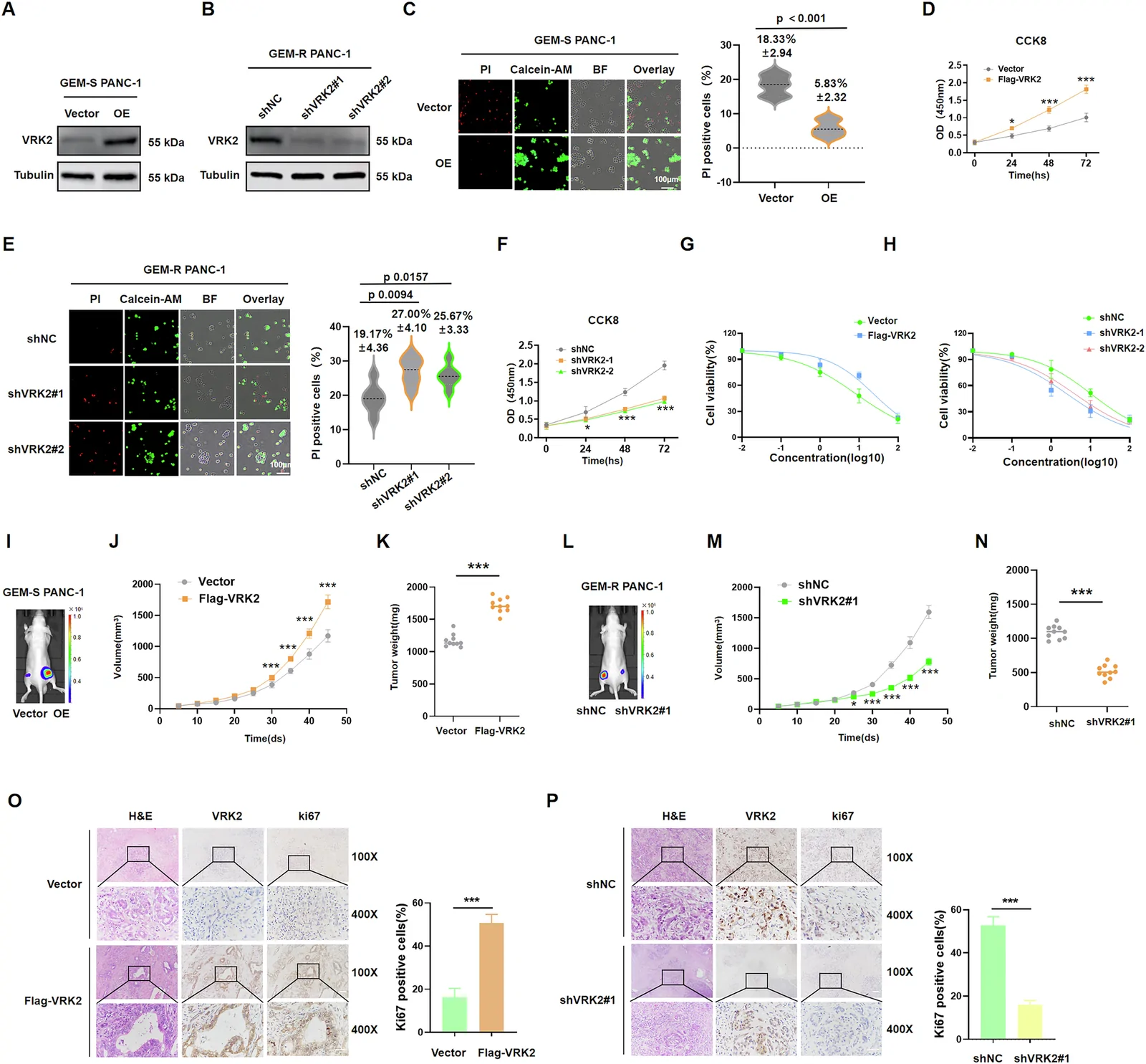
VRK2 promotes the GEM resistance in vitro and in vivo. **A, B** VRK2 overexpression in GEM-sensitive PANC-1 cells and VRK2 knockdown in GEM-resistant PANC-1 cells were performed under corresponding GEM conditions (5 μM and 2 μM, respectively), with the efficiency of genetic manipulation confirmed via Western blotting. **C, E** Calcein-AM/PI double staining was used to evaluate PANC-1 cell viability following VRK2 overexpression or knockdown in the presence of differential GEM concentrations (5 μM for GEM-resistant cells and 2 μM for GEM-sensitive cells). **D, F** Time-dependent cell viability of PANC-1 cells was assessed via CCK-8 assay upon VRK2 overexpression or knockdown, with sustained GEM treatment at line-specific concentrations (2 μM for GEM-sensitive cells and 5 μM for GEM-resistant cells). *n* = 3; *p < 0.05; ***p < 0.001. **G, H** The IC50 values of PANC-1 cells following VRK2 overexpression or knockdown in the presence of differential GEM concentrations determined by CCK8 assay (*n* = 5 for GEM-sensitive cells and *n* = 5 for GEM-resistant cells); Vector VS Flag-VRK2: 8.42(6.71-10.61) VS 23.8(10.35-30.85) p <0.001. shNC VS shVRK2-1 VS shVRK2-2: 10.73(8.48-13.61) VS 2.61(1.73-4.01) VS 4.13 (3.30-5.68) *p* <0.001. **I** Mice injected with luciferase-expressing vector or VRK2-overexpressing GEM-sensitive PANC-1 cells were assessed by IVIS imaging system. **J** Volume of tumors in the mice with vector or VRK2-overexpressing GEM-sensitive PANC-1 cells. Tumor volumes are presented as the mean ± SD, *n* = 6; ***p < 0.001. **K** Weight of tumor in the mice with vector or VRK2-overexpressing GEM-sensitive PANC-1 cells. *n* = 6; ***p < 0.001. **L** Mice injected with luciferase-expressing shNC or shVRK2 GEM-resistant PANC-1 cells were assessed by IVIS imaging system. **M** Volume of tumors in the mice with shNC or shVRK2 GEM-resistant PANC-1 cells. Tumor volumes are presented as the mean ± SD, *n* = 6; *p < 0.05; ***p < 0.001. **N** Weight of tumor in the mice with shNC or shVRK2 GEM-resistant PANC-1 cells. *n* = 6; ***p < 0.001. **O** IHC analysis of VRK2, Ki67 expression of tumors in the mice with vector or VRK2-overexpressing GEM-sensitive PANC-1 cells. *n* = 6; ***p < 0.001. For 100×figures, the scale bar indicates 100 μm; For 400×figures, the scale bar indicates 50 μm. **P** IHC analysis of VRK2, Ki67 expression of tumors in the mice with shNC or shVRK2 GEM-resistant PANC-1 cells. *n* = 6; ***p < 0.001. For 100×figures, the scale bar indicates 100 μm; For 400×figures, the scale bar indicates 50 μm.

To explore the functional role of VRK2 in mediating GEM resistance in PC, we upregulated VRK2 expression in GEM-sensitive PC cells (Fig. 2A and Supplementary Fig. 2A) and knocked down VRK2 expression in GEM-resistant PC cells (Fig.2B and Supplementary Fig. 2B). Results from Calcein-AM/PI double staining and CCK-8 assays demonstrated that, in the presence of GEM, VRK2 overexpression reduced GEM-induced cell death in GEM-sensitive PC cells, whereas VRK2 knockdown increased GEM-induced cell death in GEM-resistant PC cells (Fig. 2C, D, E, F and Supplementary Fig. 2C, D, E, F). To further validate the impact of VRK2 on GEM sensitivity, we generated dose-response curves and calculated the IC50 values. VRK2 overexpression in sensitive cells resulted in a significant increase in GEM IC50, while VRK2 knockdown in resistant cells led to a notable decrease in GEM IC50 (Fig. 2G, H and Supplementary Fig. 2G, H), confirming VRK2’s regulatory role in GEM resistance. To investigate the role of VRK2 in GEM resistance in vivo, we established xenograft models by subcutaneously implanting nude mice with either GEM-sensitive PC cells stably overexpressing VRK2 or GEM-resistant PC cells expressing a VRK2 interference vector, followed by intraperitoneal GEM treatment. Consistent with in vitro observations, VRK2 overexpression significantly enhanced tumor growth, as indicated by increased volume and weight, under GEM treatment (Fig. 2I–K and Supplementary Fig. 2I–K). Conversely, VRK2 knockdown in resistant cells substantially inhibited tumor progression, clearly demonstrating VRK2’s critical role in regulating GEM sensitivity in PC (Fig. 2L–N and Supplementary Fig. 2L–N). IHC analysis revealed that VRK2 overexpression significantly upregulated the Ki-67 positive rate in GEM-sensitive PC tissues, whereas VRK2 knockdown markedly reduced the Ki-67 positivity in GEM-resistant PC tissues following GEM treatment (Fig. 2O, P and Supplementary Fig. 2O, P). Collectively, these findings indicated that VRK2 endowed PC cells with GEM resistance in vitro and in vivo.

#### Aerobic glycolysis is involved in VRK2-mediated GEM resistance in PC

**Fig. 3:**
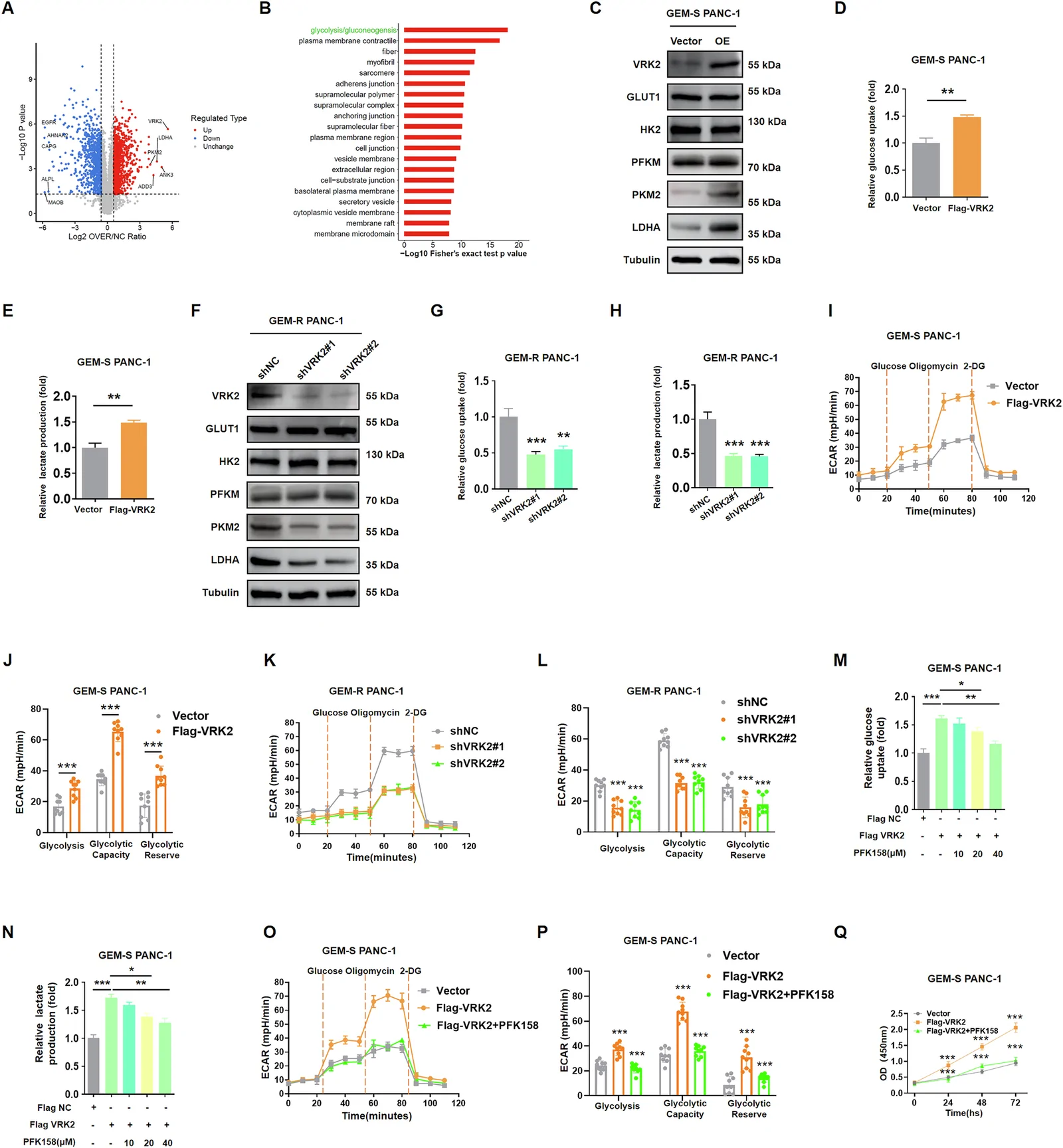
Aerobic glycolysis is involved in VRK2-mediated GEM resistance in PC. **A** Volcano plot for differentially expressed proteins following VRK2 overexpression in GEM-sensitive PANC-1 cells under GEM treatment (2 μM). **B** GO analysis for the genes enriched in the top-ranked list following VRK2 overexpression in the presence of GEM (2 μM). **C** Immunoblot for protein expression of GLUT1, HK2, PFKM, PKM2 and LDHA in VRK2-overexpressing GEM-sensitive PANC-1 cells treated with 2 μM GEM. **D, E** Relative levels of glucose uptake and lactate production in VRK2-overexpressing GEM-sensitive PANC-1 cells treated with 2 μM GEM. *n* = 3; **p < 0.01. **F** Immunoblot for protein expression of GLUT1, HK2, PFKM, PKM2 and LDHA in VRK2-knockdown GEM-resistant PANC-1 cells treated with 5 μM GEM. **G, H** The relative levels of glucose uptake and lactate production of GEM-resistant PANC-1 cells with VRK2 knockdown in the presence of GEM (5 μM). *n* = 3; **p < 0.01; ***p < 0.001. **I, J** The effect of VRK2 overexpression on ECAR in GEM-sensitive PANC-1 cells treated with 2 μM GEM. *n* = 3; ***p < 0.001. **K, L** The effect of VRK2 knockdown on ECAR in VRK2-knockdown GEM-resistant PANC-1 cells treated with 5 μM GEM. *n* = 3; ***p < 0.001. **M, N** The relative levels of glucose uptake and lactate production in VRK2-overexpressing GEM-sensitive PANC-1 cells treated with 2 μM GEM in the presence or absence of PFK158(30 μM). *n* = 3; *p < 0.05; **p < 0.01; ***p < 0.001. **O, P** The effect of VRK2 overexpression on ECAR in GEM-sensitive PANC-1 cells treated with 2 μM GEM in the presence or absence of PFK158(30 μM). *n* = 3; *p < 0.05; **Q** Effects of VRK2 overexpression on cell viability in PANC-1 cells exposed to 2 μM GEM with or without PFK158(30 μM). *n* = 3; ***p < 0.001.

To determine the mechanism through which VRK2 promotes GEM resistance in PC, we performed proteomic mass spectrometry analysis on GEM-sensitive PC cells with VRK2 upregulation in the presence of GEM (Fig. 3A). Pathway enrichment and Gene Ontology (GO) analyses revealed that the proteomic expression changes induced by VRK2 upregulation predominantly involved genes associated with glycolysis and gluconeogenesis (Fig. 3B). We next detected the expression of key glycolytic molecules under sustained GEM treatment, and found that VRK2 upregulation elevated the expression of pyruvate kinase isozyme type M2(PKM2) and lactate dehydrogenase A(LDHA) but not the expression of glucose transporter 1(GLUT1), hexokinase 2(HK2) or phosphofructokinase, muscle(PFKM) (Fig. 3C and Supplementary Fig. 3A). Under GEM exposure, VRK2-upregulated GEM-sensitive PC cells exhibited increased glucose uptake and lactate production relative to the control group (Fig. 3D, E and Supplementary Fig. 3B, C). On the contrary, VRK2 knockdown in GEM-resistant PC cells not only inhibited the expression of PKM2 and LDHA, but also promoted a decrease in glucose uptake and lactate production (Fig. 3F–H and Supplementary Fig. 3D–F). In line with these results, extracellular acidification rate (ECAR) was prominently enhanced in VRK2-overexpressing cells upon GEM stimulation, whereas VRK2 depletion yielded the opposite phenotype (Fig. 3I–L and Supplementary Fig. 3G–J). To establish a causal relationship between VRK2-induced glycolysis and GEM resistance, we treated cells with PFK158, a highly selective small-molecule inhibitor of the key glycolytic regulator PFKFB3. PFK158 intervention efficiently reversed the VRK2-mediated increases in glucose uptake, lactate secretion and ECAR (Fig. 3M–P and Supplementary Fig. 3K–N). Notably, PFK158 also inhibited the pro-survival effect of VRK2 overexpression in the presence of GEM (Fig. 3Q and Supplementary Fig. 3O). Therefore, these results suggested VRK2 facilitates GEM resistance by reinforcing aerobic glycolysis.

#### VRK2 promotes glycolysis and GEM-resistance via TPI1 in PC

**Fig. 4:**
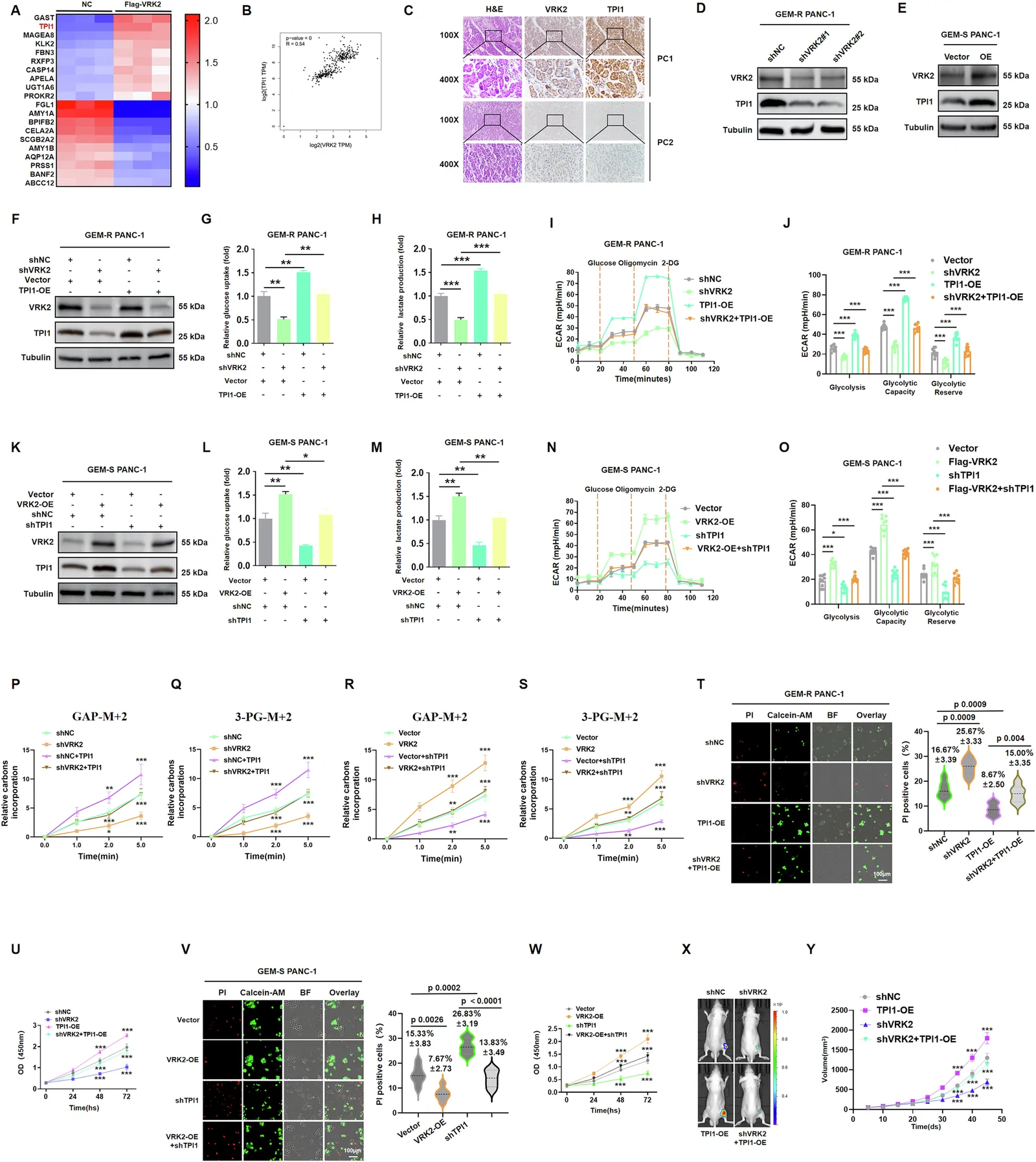
VRK2 promotes glycolysis and GEM-resistance via TPI1 in PC. **A** Heatmap for the genes expressed in the top-ranked list following VRK2 overexpression in the presence of GEM (2 μM). **B** A positive correlation between VRK2 and TPI1 expression was revealed by GEPIA analysis. **C** IHC analysis of VRK2 and TPI1 expression in tumor tissues from PC patients. For 100×figures, the scale bar indicates 100 μm; For 400×figures, the scale bar indicates 50 μm. **D** Immunoblot for protein expression of TPI1 in VRK2-knockdown GEM-resistant PANC-1 cells treated with 5 μM GEM. **E** Immunoblot for protein expression of TPI1 in VRK2-overexpressing GEM-sensitive PANC-1 cells treated with 2 μM GEM. **F** The expression of TPI1 in VRK2-knockdown GEM-resistant PANC-1 cells in the presence of GEM (5 μM) without or with TPI1 overexpression. **G, H** The relative levels of glucose uptake and lactate production in VRK2-knockdown GEM-resistant PANC-1 cells in the presence of GEM (5 μM) without or with TPI1 overexpression. *n* = 3; **p < 0.01; ***p < 0.001. **I, J** ECAR in VRK2-knockdown GEM-resistant PANC-1 cells in the presence of GEM (5 μM) without or with TPI1 overexpression. *n* = 3; ***p < 0.001. **K** The expression of TPI1 in VRK2-overexpressing GEM-sensitive PANC-1 cells in the presence of GEM (2 μM) without or with TPI1 knockdown. **L, M** The relative levels of glucose uptake and lactate production in VRK2-overexpressing GEM-sensitive PANC-1 cells in the presence of GEM (2 μM) without or with TPI1 knockdown. *n* = 3; *p < 0.05; **p < 0.01. **N, O** ECAR in VRK2-overexpressing GEM-sensitive PANC-1 cells in the presence of GEM (2 μM) without or with TPI1 knockdown. *n* = 3; *p < 0.05; ***p < 0.001. **P, Q** Carbons (M + 2) enrichment in GAP and 3-PG in VRK2-knockdown GEM-resistant PANC-1 cells in the presence of GEM (5 μM) without or with TPI1 overexpression. *n* = 3; **p < 0.01; ***p < 0.001. **R, S** Carbons (M + 2) enrichment in GAP and 3-PG in VRK2-overexpressing GEM-sensitive PANC-1 cells in the presence of GEM (2 μM) without or with TPI1 knockdown. *n* = 3; **p < 0.01; ***p < 0.001. **T, U** The viability of 5 μM GEM-exposed PANC-1 cells following VRK2 knockdown with or without TPI1 overexpression was evaluated by Calcein-AM/PI double staining and CCK-8 assay. *n* = 3; ***p < 0.001. **V, W** The viability of 2 μM GEM-exposed PANC-1 cells following VRK2 overexpression without or with TPI1 knockdown was tested by calcein-AM/PI double staining and CCK8 assay. *n* = 3; ***p < 0.001. **X** Following GEM intervention, in vivo bioluminescence of mice inoculated with GEM-resistant PANC-1 cells expressing luciferase and shNC/shVRK2, with or without TPI1 overexpression, was detected via the IVIS imaging system. **Y** The tumor volume of mice implanted with GEM-resistant PANC-1 cells with distinct genetic modifications was evaluated upon GEM intervention. Tumor volumes are presented as the mean ± SD, *n* = 6; ***p < 0.001.

To probe the downstream target of VRK2 that controls glycolysis and GEM-resistance, we carried out transcriptome sequencing in VRK2-upregulated GEM-sensitive PC cells cultured under steady GEM exposure. Triosephosphate isomerase (TPI1), a core glycolytic enzyme that catalyzes the reversible isomerization of triosephosphate to facilitate the interconversion between dihydroxyacetone phosphate (DHAP) and glyceraldehyde-3-phosphate (GAP), was markedly increased in VRK2-overexpressing cells (Fig. 4A). Gene expression correlation analysis via the GEPIA database confirmed a positive linear relationship between VRK2 and TPI1 expression in clinical PC tissues (Fig. 4B). Consistently, IHC staining revealed that PC tissues with elevated expression of VRK2 frequently displayed increased expression of TPI1 (Fig. 4C). Moreover, within a GEM-exposed culture context, TPI1 was downregulated in VRK2-knockdown GEM-resistant PC cells, whereas its expression was significantly elevated in VRK2-overexpressing GEM-sensitive cells (Fig. 4D, E and Supplementary Fig. 4A, B). Collectively, these data support that TPI1 serves as a bona fide downstream target of VRK2.

We next sought to determine whether VRK2 drives glycolytic reprogramming and GEM resistance via modulating TPI1 expression. All subsequent rescue and functional verification experiments were conducted in the presence of GEM to recapitulate chemotherapeutic stress. We first increased TPI1 expression in VRK2-knockdown cells and found that the downregulation of VRK2 inhibited TPI1 expression, whereas TPI1 upregulation impaired the decline in TPI1 expression caused by VRK2 knockdown (Fig. 4F and Supplementary Fig. 4C). TPI1 overexpression rescued the reduction of glucose uptake, lactate production and ECAR induced by downregulation of VRK2 expression (Fig. 4G–J and Supplementary Fig. 4D–G). In contrast, VRK2 overexpression enhanced TPI1 expression, while TPI1 knockdown counteracted VRK2-induced increase in the expression of TPI1 (Fig. 4K and Supplementary Fig. 4H). Similarly, the enhanced glycolytic activity induced by VRK2 overexpression was profoundly compromised upon TPI1 knockdown (Fig. 4L–O and Supplementary Fig. 4I–L). These results demonstrate that VRK2 potentiates glycolytic flux in PC cells in a TPI1-dependent manner.

To further delineate the connection between VRK2-mediated regulation of TPI1 expression and anaerobic glycolysis, we employed positionally-labeled 1,2-^13^C_2_ glucose-based metabolic flux analysis (MFA) to quantify the DHAP/GAP interconversion flux under low-glucose conditions (0.5 mM) at 1-, 2-, and 5-minute intervals [11]. This isotopic labeling technique enables precise evaluation of glucose-derived carbon flux through the glycolytic pathway by tracking isotopic enrichment patterns in key metabolic intermediates. Notably, TPI1 enzymatic activity can be effectively assessed by monitoring M + 2 labeled carbon incorporation into GAP and downstream glycolytic intermediates, as GAP directly derived from F-1,6-BP remains unlabeled (M + 0), whereas GAP converted from DHAP via TPI1-mediated reaction exhibits M + 2 labeling. Additionally, GAP derived from 6-PG displays single-carbon labeling (M + 1). As demonstrated in Fig. 4P, Q and Supplementary Fig. 4M–O, TPI1 overexpression robustly reversed the VRK2 knockdown-induced decline in carbons (M + 2) enrichment in GAP and 3-phosphoglycerate (3-PG) at 2- and 5-minute time points. Conversely, TPI1 knockdown significantly attenuated the increased carbons (M + 2) incorporation into these glycolytic intermediates mediated by VRK2 overexpression (Fig. 4R, S and Supplementary Fig. 4P, Q). Additionally, calcein-AM/PI double staining and CCK-8 assays under GEM exposure validated the indispensable role of TPI1 in VRK2-mediated chemoresistance. TPI1 overexpression efficiently mitigated GEM sensitization in VRK2-knockdown cells, whereas TPI1 knockdown compromised the chemoresistant phenotype conferred by VRK2 overexpression (Fig. 4T–W and Supplementary Fig. 4R–U). These results indicated that TPI1 was a key protein for VRK2-induced glycolysis and GEM-resistance in PC in vitro.

Consistent with in vitro experimental settings, our in vivo xenograft models were also subjected to standardized GEM treatment. In vivo studies revealed that TPI1 restoration blunted the impaired tumor growth caused by VRK2 knockdown (Fig. 4X, Y). TPI1 inhibition effectively alleviated the augmented tumor growth in VRK2-overexpressing xenografts (Supplementary Fig. 4V, W). Taken together, our in vitro and in vivo evidence demonstrates that TPI1 is functionally regulated by VRK2 and acts as an essential mediator to promote aerobic glycolysis and confer GEM resistance in PC.

### SP1 is identified as a transcriptional factor regulating the expression of TPI1

**Fig. 5:**
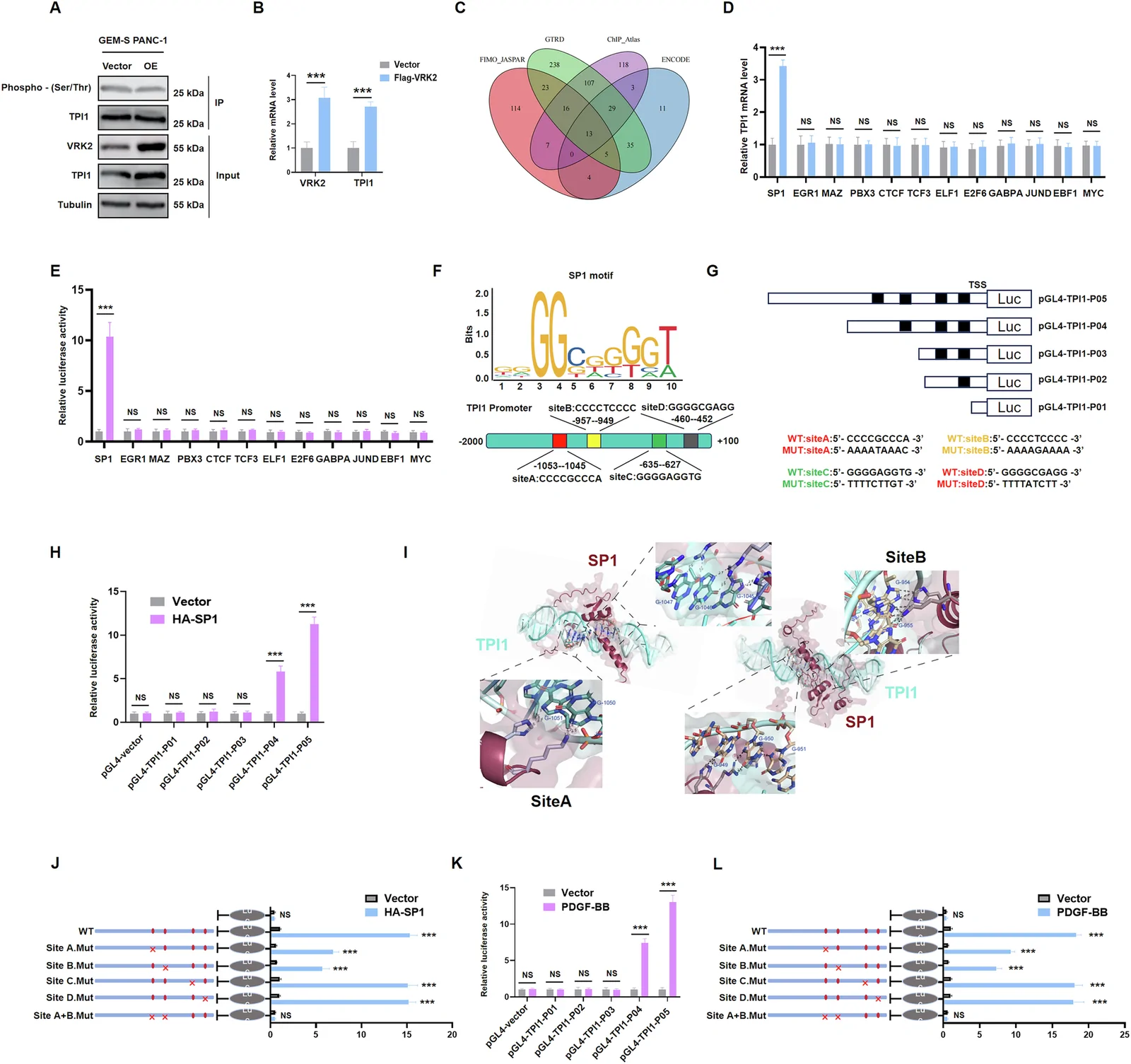
SP1 is identified as a transcriptional factor regulating the expression of TPI1. **A** Immunoblot for protein expression of p-TPI1 and TPI1 in VRK2-overexpressing GEM-sensitive PANC-1 cells treated with 2 μM GEM. **B** The relative mRNA level of TPI1 in VRK2-overexpressing GEM-sensitive PANC-1 cells treated with 2 μM GEM. *n* = 3; ***p < 0.001. **C** Potential transcription factors regulating TPI1 expression predicted by FIMO_JASPAR, GTRD, ChIP_Atlas and ENCODE databases. **D** Relative mRNA levels of TPI1 in PANC-1 cells with potential transcription factors overexpression. *n* = 3; NS, no significant; ***p < 0.001. **E** Relative transcriptional activity of TPI1 in PANC-1 cells with potential transcription factors overexpression. *n* = 3; NS, no significant; ***p < 0.001. **F** Top, the sequence logo and frequency matrix of SP1 obtained from the JASPAR website. Bottom, schematic of the four most potent SP1-binding sites in the promotor region of the TPI1 gene. **G** Top, schematic of truncated constructs containing SP1 binding regions in the TPI1 promotors. Bottom, sequences of the wild-type (WT) and mutated (MUT) binding sites in the four promotor regions used in the luciferase reporter constructs. **H** The relative transcriptional activity of TPI1 in PANC-1 cells co-transfected with an SP1 expression plasmid and truncated constructs containing SP1-binding regions within the TPI1 promoter. *n* = 3; NS, no significant; ***p < 0.001. **I** Dynamic structural analysis for SP1-binding sites in the promotor region of the TPI1 gene. **J** Relative luciferase activities of different reporters containing wild-type (WT) and mutated (MUT) sequences of the TPI1 promotor in PANC-1 cells expressing SP1 plasmid. Filled circles represent the putative SP1-binding sites, whereas ‘**×**’ marks the mutated SP1-binding sites. *n* = 3; NS, no significant; ***p < 0.001. **K** The relative transcriptional activity of TPI1 in PANC-1 cells co-transfected with PDGF-BB expression plasmid and truncated constructs containing SP1-binding regions within the TPI1 promoter. *n* = 3; NS, no significant; ***p < 0.001. **L** Relative luciferase activities of different reporters containing wild-type (WT) and mutated (MUT) sequences of the TPI1 promotor in PANC-1 cells expressing PDGF-BB plasmid. Filled circles represent the putative SP1-binding sites, whereas ‘×’ marks the mutated SP1-binding sites. *n* = 3; NS, no significant; ***p < 0.001.

Given that VRK2 acts as a protein kinase, we first explored whether VRK2 modulates TPI1 expression through direct TPI1 phosphorylation. Western blot results showed that VRK2 overexpression exerted no obvious effect on the phosphorylation status of TPI1 (Fig. 5A and Supplementary Fig. 5A). However, TPI1 mRNA levels were markedly elevated in VRK2-overexpressing PC cells cultured under GEM exposure (Fig. 5B and Supplementary Fig. 5B), suggesting that VRK2 upregulates TPI1 at the transcriptional level. We next sought to identify the key transcription factors responsible for TPI1 regulation. Bioinformatic prediction using FIMO_JASPAR, GTRD, ChIP_Atlas and ENCODE databases screened 13 candidate transcription factors capable of binding the TPI1 promoter region (Fig. 5C). Among these candidates, 12 showed significantly positive expression correlations with TPI1 in GEPIA clinical dataset analysis (Supplementary Fig. 5C). We further performed qRT-PCR and luciferase reporter assays to verify their regulatory roles, and the results demonstrated that only SP1 effectively elevated TPI1 transcription and promoter reporter activity (Fig. 5D, E and Supplementary Fig. 5D, E). Four conserved SP1-binding motifs were predicted within the human TPI1 promoter, approximately 2000 bp upstream of the transcription start site (TSS) (Fig. 5F). To functionally characterize these sites, we engineered a series of truncated promoter constructs (Fig. 5G) and performed luciferase reporter assays upon co-transfection with SP1 expression plasmid. Notably, constructs containing Site A ( − 1053 to −1045) and Site B ( − 957 to −949) exhibited robust transcriptional activation upon SP1 overexpression (Fig. 5H and Supplementary Fig. 5F). Structural simulation analysis further confirmed that Sites A and B were high-confidence SP1-binding loci (Fig. 5I). Subsequent site-directed mutagenesis demonstrated that individual mutations at either Site A or Site B significantly attenuated luciferase activity, with combined mutations producing synergistic suppression (Fig. 5J and Supplementary Fig. 5G). Functional validation using PDGF-BB, a known SP1 signaling activator, confirmed that Sites A and B mediated SP1-dependent transcriptional enhancement (Fig. 5K and Supplementary Fig. 5H), while their mutations abrogated PDGF-BB responsiveness (Fig. 5L and Supplementary Fig. 5I). In contrast, mutations at Sites C (–635 to –627) and Site D (–460 to –452) showed no significant effects across all experimental conditions. Together, these data suggest that SP1 plays an important role in mediating TPI1 expression in PC cells.

### VRK2 phosphorylates SP1 at Ser2 and promotes its nuclear import

**Fig. 6:**
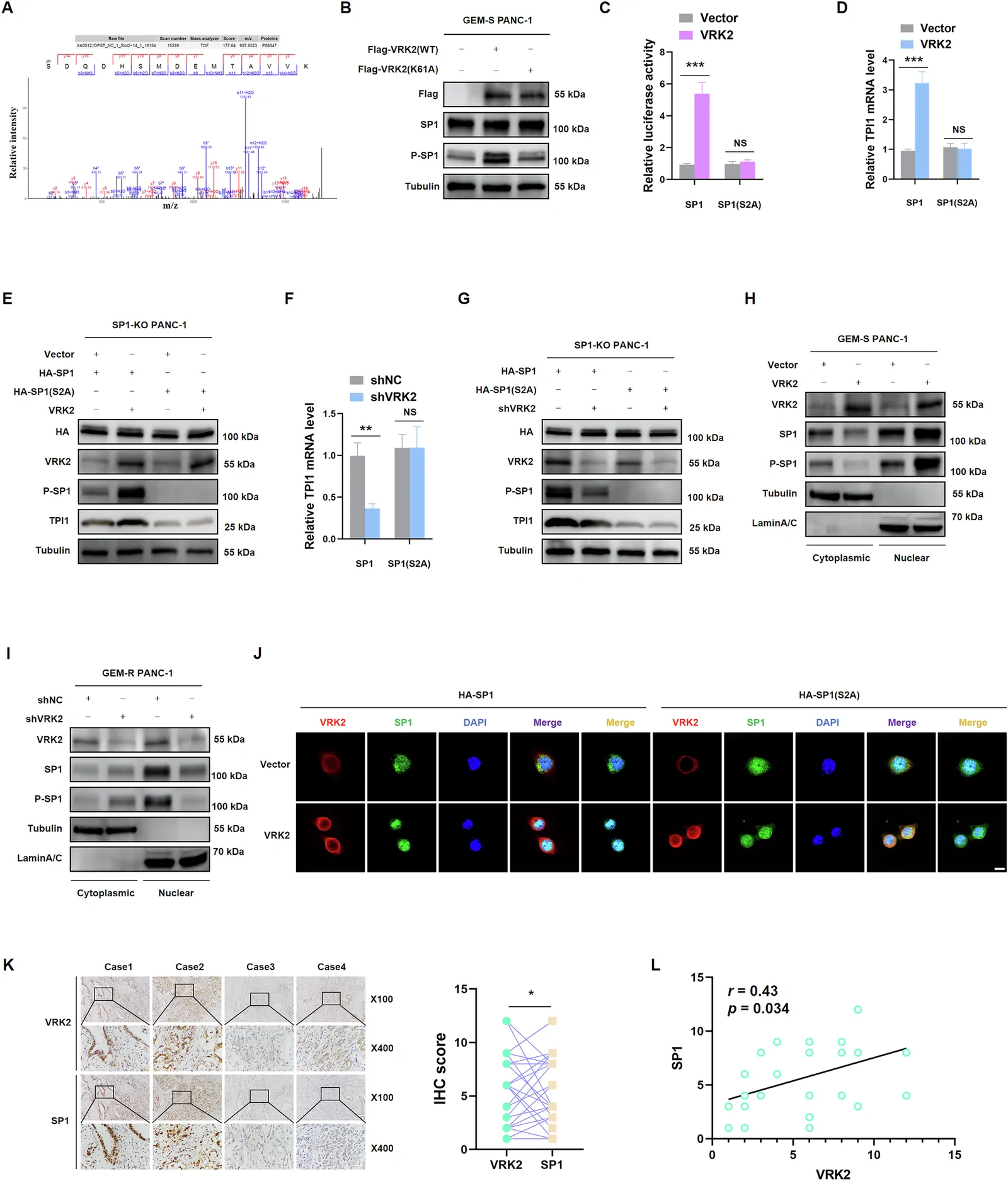
VRK2 phosphorylates SP1 at Ser2 and promotes its nuclear import. **A** Mass spectrometry analysis for the substrates‌ of kinase VRK2. **B** Western blot analysis of p-SP1 and total SP1 expression in PANC-1 cells harbouring wild-type VRK2 or kinase-dead VRK2 (K61A) following 2 μM GEM stimulation. **C** The effect of VRK2 on TPI1 transcriptional activity in SP1-knockout PANC-1 cells reconstituted with wild-type SP1 or SP1 (S2A) mutant plasmids under 2 μM GEM treatment. *n* = 3; NS, no significant; ***p < 0.001. **D, E** The effect of VRK2 on TPI1 mRNA and protein levels in SP1-knockout PANC-1 cells reconstituted with wild-type SP1 or SP1 (S2A) mutant plasmids upon 2 μM GEM treatment. *n* = 3; NS, no significant; ***p < 0.001. **F, G** TPI1 mRNA and protein levels regulated by VRK2 knockdown in SP1-knockout PANC-1 cells reconstituted with wild-type SP1 or SP1 (S2A) mutant plasmids following 5 μM GEM treatment. *n* = 3; NS, no significant; **p < 0.01. **H, I** Cytoplasmic and nuclear protein levels of SP1 and p-SP1 in VRK2-overexpressing or VRK2-knockdown PANC-1 cells under prolonged GEM treatment at cell line-specific concentrations (2 μM for GEM-sensitive cells and 5 μM for GEM-resistant cells). **J** Modulation of SP1 subcellular localization by VRK2 in SP1-deficient PANC-1 cells reconstituted with wild-type SP1 or S2A-mutant SP1 under 2 μM GEM exposure. Scale bar indicates 20 μm. **K** IHC analysis of VRK2 expression and subcellular localization of SP1 in human PC tissues. *p < 0.05. For 100×figures, the scale bar indicates 100 μm; For 400×figures, the scale bar indicates 50 μm. **L** Correlation of VRK2 levels with nuclear SP1 expression in human PC tissues.

Phosphorylation-mediated post-translational modification constitutes an evolutionarily conserved mechanism through which cells spatiotemporally modulate transcription factor functionality, including nuclear translocation, DNA-binding affinity, and transactivation potential [22]. Intriguingly, mass spectrometry analysis identified transcription factor SP1 as a ‌novel substrate‌ of kinase VRK2, with Ser2 identified as a high-confidence phosphorylation site (Fig. 6A). Enhanced phosphorylation of SP1 at Ser2 by wild-type VRK2 was confirmed via Western blot analysis, whereas no such enhancement was observed with the kinase-dead VRK2 (K61A) mutant, which was generated through a Lys61-to-alanine substitution at the catalytic residue (Fig. 6B and Supplementary Fig. 6A). To determine whether SP1 Ser2 phosphorylation is required for TPI1 transcription, SP1-knockout PC cells were reconstituted with wild-type SP1 or the phosphorylation-deficient SP1(S2A) mutant. Luciferase assays showed that VRK2 enhanced TPI1 promoter activity in cells re-expressing wild-type SP1, but not in SP1(S2A)-restored cells (Fig. 6C and Supplementary Fig. 6B). Consistently, VRK2-mediated upregulation of TPI1 mRNA and protein was observed only in SP1-knockout cells with wild-type SP1 reconstitution (Fig. 6D, E and Supplementary Fig. 6C, D). Reciprocally, VRK2 knockdown reduced TPI1 expression in wild-type SP1-rescued cells, yet caused no obvious changes in cells expressing SP1(S2A) (Fig. 6F, G and Supplementary Fig. 6E, F). These findings confirm that VRK2-catalyzed SP1 phosphorylation at Ser2 is essential for TPI1 transcriptional activation.

Given established links between transcription factor phosphorylation and subcellular translocation [23], we further detected the influence of VRK2 on SP1 nuclear distribution under routine GEM culture conditions. VRK2 overexpression increased SP1 Ser2 phosphorylation and nuclear accumulation, while VRK2 knockdown reduced both events (Fig. 6H, I and Supplementary Fig. 6G, H). Importantly, VRK2-dependent SP1 nuclear translocation was observed exclusively in cells reconstituted with wild-type SP1, rather than the SP1(S2A) mutant (Fig. 6J and Supplementary Fig. 6I). In clinical PC tissues, high VRK2 expression was accompanied by elevated nuclear SP1 abundance, and a positive correlation between VRK2 and nuclear SP1 levels was verified (Fig. 6K, L). Collectively, VRK2 phosphorylates SP1 at Ser2 to facilitate SP1 nuclear translocation, thereby initiating robust TPI1 transcription.

#### The phosphorylation of SP1 is indispensable for VRK2-triggered TPI1 expression, glycolysis and GEM-resistance

**Fig. 7:**
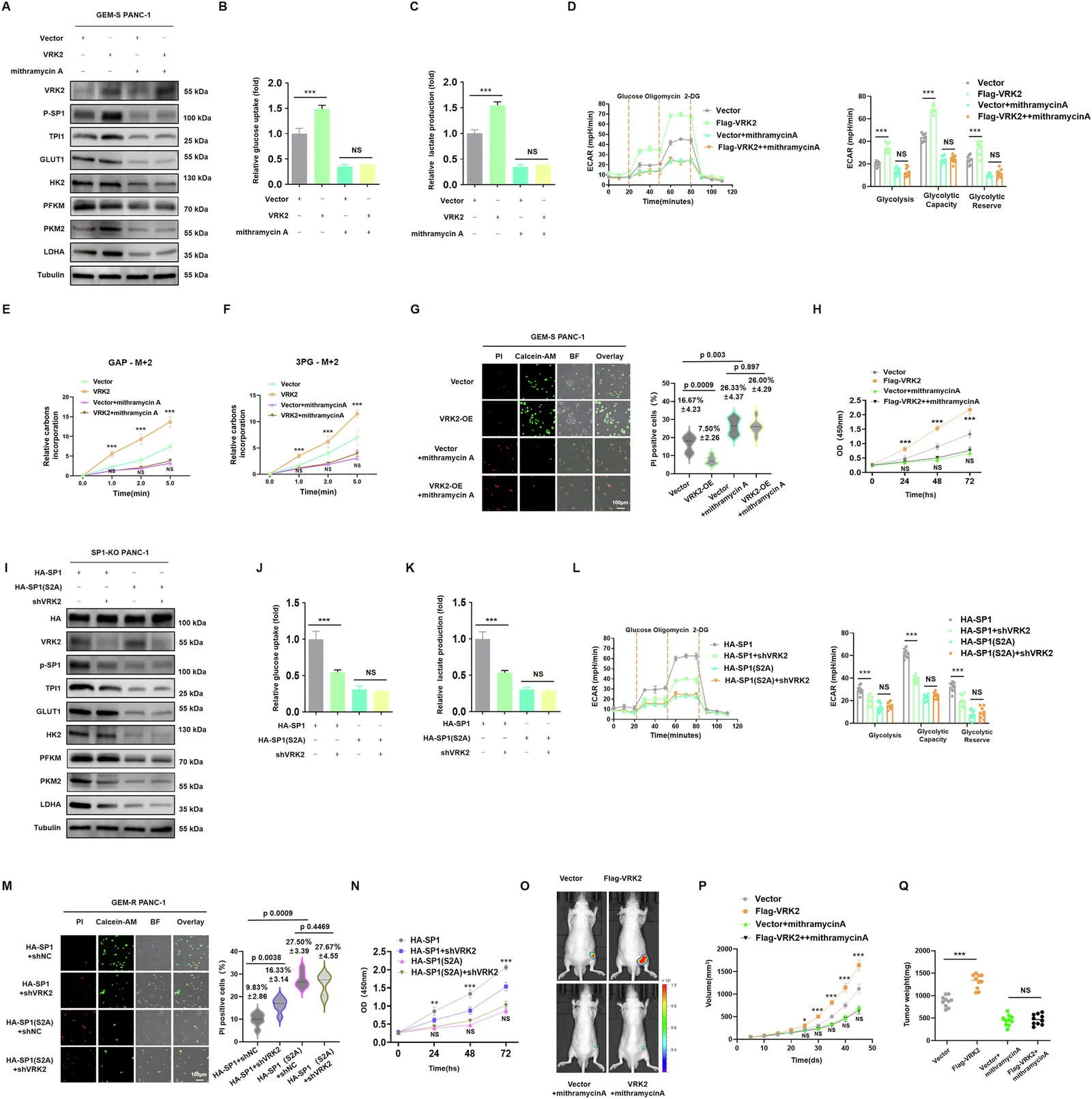
The phosphorylation of SP1 is indispensable for VRK2-triggered TPI1 expression, glycolysis and GEM-resistance. **A** Immunoblot analysis of p-SP1, TPI1, GLUT1, HK2, PFKM, PKM2 and LDHA protein levels in VRK2-overexpressing PANC-1 cells treated with 2 μM GEM, with or without 200 nM mithramycin A intervention for 24 h. **B, C** Relative glucose uptake and lactate production levels in VRK2-overexpressing PANC-1 cells treated with 2 μM GEM, with or without 200 nM mithramycin A treatment for 24 h. *n* = 3; NS, no significant; ***p < 0.001. **D** ECAR levels in VRK2-overexpressing GEM-sensitive PANC-1 cells treated with 2 μM GEM, with or without 200 nM mithramycin A treatment for 24 h. *n* = 3; NS, no significant; ***p < 0.001. **E, F** Carbons (M + 2) enrichment in GAP and 3-PG in VRK2-overexpressing GEM-sensitive PANC-1 cells treated with 2 μM GEM, with or without 200 nM mithramycin A treatment for 24 h. *n* = 3; ***p < 0.001. **G, H** Cell viability of VRK2-overexpressing PANC-1 cells treated with 2 μM GEM, with or without 200 nM mithramycin A treatment for 24 h, detected via Calcein-AM/PI double staining and CCK-8 assay. *n* = 3; NS, no significant; ***p < 0.001. **I** The effects of VRK2 knockdown on the protein levels of p-SP1, TPI1, GLUT1, HK2, PFKM, PKM2, and LDHA in SP1-knockout PANC-1 cells reconstituted with wild-type SP1 or SP1(S2A) mutant plasmids under 5 μM GEM treatment. **J, K** The effects of VRK2 knockdown on glucose uptake and lactate production in SP1-knockout PANC-1 cells reconstituted with wild-type SP1 or SP1(S2A) mutant plasmids upon 5 μM GEM exposure. *n* = 3; NS, no significant; ***p < 0.001. **L** The effects of VRK2 knockdown on ECAR in SP1-knockout PANC-1 cells reconstituted with wild-type SP1 or SP1(S2A) mutant plasmids under 5 μM GEM treatment. *n* = 3; NS, no significant; ***p < 0.001. **M, N** Calcein-AM/PI double staining and CCK8 assays showing the effects of VRK2 knockdown on the viability of SP1-knockout PANC-1 cells reconstituted with wild-type SP1 or SP1(S2A) mutant plasmids under 5 μM GEM treatment. *n* = 3; NS, no significant; **p < 0.01; ***p < 0.001. **O** Mice injected with luciferase-labeled control or VRK2-overexpressing GEM-sensitive PANC-1 cells were subjected to GEM treatment with or without mithramycin A, followed by IVIS imaging analysis. **P, Q** Tumor volume and weight in mice inoculated with GEM-sensitive PANC-1 cells transfected with vector, VRK2-overexpressing construct, vector + mithramycin A, or VRK2-overexpressing construct + mithramycin A, followed by GEM treatment. Tumor volumes are presented as the mean ± SD, *n* = 6; NS, no significant; *p < 0.05; ***p < 0.001.

Having demonstrated that SP1 acts as a direct phosphorylated substrate of VRK2 and a key upstream activator of TPI1, we next investigated whether SP1 phosphorylation contributes to VRK2-driven glycolytic reprogramming and chemoresistance under GEM exposure. VRK2-overexpressing PC cells were treated with mithramycin A, a selective SP1 activity inhibitor. Mithramycin A markedly suppressed endogenous SP1 function and blocked the elevation of SP1 phosphorylation and TPI1 expression induced by VRK2 overexpression (Fig. 7A). Moreover, SP1 inhibition completely abolished the increases in glucose uptake, lactate production and ECAR triggered by VRK2 (Fig. 7B–D). Metabolic flux analysis further showed that the enhanced M + 2-labeled glycolytic carbon flux toward GAP and 3-PG in VRK2-overexpressing cells was abrogated upon mithramycin A treatment (Fig. 7E, F). Functional cell assays performed under GEM treatment further validated that mithramycin A efficiently reversed VRK2-conferred chemoresistance (Fig. 7G, H).

In subsequent rescue experiments, VRK2 was downregulated in SP1-knockout cells reconstituted with wild-type SP1 or SP1(S2A). Under GEM culture conditions, VRK2 downregulation reduced SP1 phosphorylation and TPI1 expression in wild-type SP1-rescued cells, but not in SP1(S2A)-expressing cells (Fig. 7I). Similarly, the glycolytic deficits caused by VRK2 knockdown, including decreased glucose uptake, lactate release and ECAR, were only evident in cells with wild-type SP1 reconstitution (Fig. 7J–L). In line with these metabolic phenotypes, VRK2 knockdown significantly lowered cell viability in response to GEM in wild-type SP1-rescued cells, while no such chemosensitizing effect was detected in SP1(S2A) groups (Fig. 7M, N). In vivo xenograft models were also established with GEM administration, and results showed that mithramycin A treatment effectively blocked VRK2-promoted tumor growth (Fig. 7O–Q). In addition, no notable alterations in serum liver and renal function biomarkers were detected across groups throughout treatment, confirming that mithramycin A poses little damage to mouse hepatic and renal function (Supplementary Fig. 7A–D). Collectively, these results demonstrate that SP1 Ser2 phosphorylation is required for VRK2-induced TPI1 upregulation, glycolytic activation and GEM resistance in PC.

## Discussion

Chemotherapeutic resistance remains a critical challenge in PC treatment, with both intrinsic cellular mechanisms and acquired adaptations contributing to therapeutic failure [24, 25]. Notably, oncogene-driven metabolic reprogramming has emerged as a key determinant of chemoresistance in malignant tumors [26]. Our study demonstrates that oncogenic VRK2 is significantly upregulated in GEM-resistant PC cells, where it promotes aerobic glycolysis to confer drug resistance. Mechanistically, VRK2 enhances TPI1 expression through a coordinated pathway involving phosphorylation of transcription factor SP1 and subsequent nuclear translocation. These findings not only elucidate the molecular basis of VRK2-mediated GEM resistance but also underscore metabolic reprogramming as a promising therapeutic target, offering translational potential for overcoming chemotherapy resistance in PC patients.

Emerging evidence has shown that metabolic reprogramming of cancer cells, especially aerobic glycolysis, is closely linked to drug resistance. On the one hand, enhanced glycolytic activity yields sufficient ATP levels to meet the elevated energy demands of cancer cells, which is consumed to implement drug efflux and consequent resistance to chemotherapeutic agents. For another, excessive lactic acid generated by aerobic glycolysis fosters stemness of cancer cells resulting in chemoresistance [27]. In this study, we found that VRK2 endowed PC cells with GEM resistance through TPI1-driven aerobic glycolysis. The function of TPI1 in diverse tumor is complicated and inconsistent. Some investigations revealed that TPI1 is upregulated and functions as an oncogene in several types of tumors, such as breast cancer, gastric cancer, and lung cancer [12, 13, 28]. Other research showed that the downregulation of TPI1 expression in hepatocellular carcinoma is linked to poor outcome [14]. Here, our work verified the increased expression and tumor-promoting effect of TPI1 promoted by VRK2 in GEM-resistant PC cells. The elevated expression of TPI1 enhanced the conversion of DHAP to GAP, thereby contributing to energy synthesis and lactic acid production through the shift of metabolic pathway from lipid production towards glycolysis. The regulation of metabolites at the intersection of glycolysis and lipid synthesis to improve the efficiency of energy generation could help to overcome metabolic stresses in response to chemotherapy and support the survival of cancer cells, which provides an explanation for the mechanism by which VRK2 promotes GEM resistance through glycolysis.

We recognize that glycolysis constitutes a complex and highly interconnected metabolic network, and TPI1 is unlikely to represent the exclusive downstream mediator through which VRK2 drives gemcitabine resistance. In line with this, our data revealed that VRK2 also modulates the expression of other glycolytic enzymes, including PKM2 and LDHA, implying that VRK2 acts on multiple regulatory nodes within the glycolytic pathway. Despite this, multiple lines of evidence firmly establish TPI1 as the key and direct downstream effector of VRK2. TPI1 was identified as one of the most significantly upregulated glycolytic genes in VRK2-overexpressing cells; VRK2 directly controls TPI1 transcription by phosphorylating SP1 and promoting its nuclear translocation; bidirectional rescue experiments further confirmed that TPI1 is both necessary and sufficient to mediate the regulatory effects of VRK2 on glycolysis and chemoresistance; and metabolic flux analysis verified that TPI1 functionally mediates the VRK2-induced redistribution of glycolytic carbon flux. It should be noted that our metabolic flux analysis was conducted under low-glucose (0.5 mM) conditions and restricted to early isotopic tracing time points (1, 2, and 5 min). While this framework was optimized to sensitively detect TPI1-mediated glycolytic flux alterations before isotopic equilibrium is reached, it may not fully reflect metabolic dynamics under more physiological glucose concentrations (5–10 mM) or prolonged tracing periods. Future investigations incorporating a gradient of glucose levels and extended tracer incubation times will be instrumental in further validating and expanding our metabolic findings. While other VRK2-targeted glycolytic enzymes may contribute cooperatively to global metabolic reprogramming, future studies employing combinatorial gene knockdown or multi-omics strategies will be required to fully characterize the broader metabolic network governed by VRK2.

Allowing for the critical role in glycolysis played by TPI1, it is of significance to determine the regulatory mechanism for TPI1 expression. However, the current documents referring to the modulation of TPI1 in cancer cells are considerably limited. TPI1 phosphorylation at Ser21 by LKB1 promotes its enzyme catalytic activity [11]. In the model of osteoarthritis, the transcription activity of TPI1 has been proven to be regulated by LDHA-mediated histone H3 lysine 18 lactylation [29]. Recently, a study showed that STAU1 recruited eIF4A3 through interacting with circ-231 to enhance mRNA translation of TPI1 and thus promoted the progression of esophageal squamous cell carcinoma [30]. These findings primarily highlight epigenetic and translational regulatory mechanisms. In contrast, our study unveils a novel transcriptional regulation pathway involving transcription factor SP1, which significantly expands the understanding of TPI1 expression modulation. We identified SP1 as a potent transcriptional activator of TPI1, with specific promoter regions (Site A: −1053 to −1045; Site B: −957 to −949) being critical for SP1-mediated luciferase activity enhancement. This discovery not only complements existing regulatory frameworks but also provides new insights into the transcriptional control of TPI1 expression in cancer cells.

Phosphorylation, one of the most important posttranslational modifications, is able to transduce cellular signals to the nucleus, which has an influence on the functional properties of transcriptional factors [31]. As a serine/threonine phosphatase, VRK2 has been found to directly phosphorylate a variety of substrate proteins and then have an impact on the malignant behaviour of cancer cells [18, 20]. Our data expands the list of substrate proteins of VRK2 by revealing a previously uncharacterized link between VRK2 and transcriptional factor SP1. We found that SP1 could be phosphorylated at Ser2 by wild-type VRK2, but not by kinase-dead mutant VRK2. Previous investigations have reported diverse functional alterations induced by different phosphorylation events of SP1, which include stability of downstream proteins, DNA binding affinity, transcriptional activity, and interaction with other proteins [32]. For example, the phosphorylation of SP1 at Thr668, Ser670, and Thr681 caused by angiotensin II treatment enhances SP1 binding to the promoter of platelet-derived growth factor-D [33]. JNK-mediated SP1 phosphorylation at Thr278 and Thr739 stabilizes SP1 expression by preventing its ubiquitination and proteasomal degradation [34]. Here, we found that the phosphorylation of SP1 at Ser2 triggered by VRK2 enhanced the nuclear translocation of SP1 and the transcriptional activity of TPI1. The dephosphorylation mutant exhibits reduced nuclear accumulation of SP1, consequently downregulating TPI1 expression. Importantly, VRK2 enhances glycolysis through nuclear SP1-dependent TPI1 transcription, ultimately conferring GEM resistance in PC cells.

In summary, we identified VRK2 as a critical oncogene that promotes glycolysis and contributes to GEM resistance in PC. Our study unveils a novel regulatory mechanism by which VRK2-mediated SP1 phosphorylation modulates TPI1 transcriptional activity. These findings highlight the clinical significance and prognostic potential of VRK2 in PC. This study provides key mechanistic insights into the regulation of PC metabolic reprogramming by the VRK2-SP1-TPI1 axis. Future screening of inhibitors targeting VRK2 may provide a promising therapeutic approach for PC therapy.

## Materials and methods

### Cell culture

The human pancreatic adenocarcinoma cell lines PANC-1(RRID: CVCL_0480) and SW1990(RRID: CVCL_1723) were purchased from the Cell Bank of Type Culture Collection of the Chinese Academy of Sciences (Shanghai, China). Cells were cultured in the DMEM media (Gibco, Grand Island, NY, USA) supplemented with 10% fetal bovine serum (Gibco, Grand Island, NY, USA). All cell culture procedures were conducted in a standard cell culture incubator maintained at 37 °C with 5% CO₂ and 95% relative humidity, while the culture medium pH was consistently maintained between 7.2 and 7.4.

### Antibodies, reagents and plasmids

Anti-VRK2 (#12946-1-AP), Anti-Beta Tubulin (#10094-2-AP), Anti-Ki-67 (#28074-1-AP), Anti-GLUT1 (#21829-1-AP), Anti-HK2 (#22029-1-AP), Anti-PFKM (#55028-1-AP), Anti-PKM2 (#15822-1-AP), Anti-LDHA (#19987-1-AP), Anti-TPI1 (#10713-1-AP), Anti-SP1(#21962-1-AP), Anti-Lamin A/C (#10298-1-AP) were purchased from proteintech (Chicago, USA). Anti-Phospho - (Ser/Thr) (#ab117253) was purchased from Abcam (Cambridge, UK). Anti-SP1(Ser2) was purchased from PTM BIO (Hangzhou, China).

Calcein/PI Cell Viability and Cytotoxicity Assay Kit (#C2015S), Cell Counting Kit-8 (CCK-8 Kit, #C0037), and Glucose uptake detection kit (S0561S) were purchased from Beyotime (Shanghai, China). Lactate Assay Kit (Colorimetric/Fluorometric) was purchased from Abcam (Cambridge, UK). 2-DG (#S4701), PFK158(#S8807) and Mithramycin A (#S8145) were purchased from Selleck (Shanghai, CHN). Lipofectamine™ 3000 Transfection Reagent (#L3000015) was purchased from ThermoFisher (MA, USA). Protein A/G PLUS-agarose (#sc-2003) was purchased from Santa Cruz Biotechnology (Dallas, TX).

VRK2 short hairpin RNA (shVRK2) plasmid, TPI1 short hairpin RNA (shTPI1) plasmid, ligated in pGV248 vector, VRK2 overexpressed plasmid, TPI1 overexpressed plasmid, PDGF-BB overexpressed plasmid, SP1 overexpressed plasmid were purchased from Shanghai GeneChem Technologies (Shanghai, China). The plasmids of VRK2 mutant, SP1 mutant and TPI1 mutant were generated by site-directed mutagenesis.

### Development of GEM-resistant PC cell lines

The drug-resistant cell line was constructed using a low-dose inductive method as follows: after determining the GEM half-maximal inhibitory concentration for PC cells, an initial dose of 20 nM GEM was added to culture flasks; 24 h later, the drug-containing medium was aspirated, cells were washed with PBS, and regular medium was replenished; upon reaching >80% confluency, subculturing was performed with either the same or a fourfold-increased drug concentration—this cycle repeated until acquired resistance met experimental requirements, requiring approximately six months. Thereafter, PC cells were persistently cultured under sustained GEM drug pressure for subsequent experimental studies.

### Organoid culture

We successfully established pancreatic tumor organoid lines using fresh tumor tissue specimens obtained from patients, with written informed consent acquired from all participants and ethical approval granted by the Institutional Review Board of The Second Affiliated Hospital, Zhejiang University. The tissue surface was rinsed with Dhank’s buffer containing 2% Penicillin-Streptomycin (P/S). Using ophthalmic scissors, the tissue was minced into 1–3 mm³ fragments. Tissue dissociation solution (primary components: Collagenase Type IV, hyaluronidase, and DNase) was added to digest the tissue at 37 °C for 35 min. The resulting enzymatic digest was filtered through a 100-μm cell strainer to remove undissociated tissue fragments. The filtrate was centrifuged at 1500 rpm for 5 min. After discarding the supernatant, the pellet was resuspended and washed with 10 mL of Dhank’s buffer containing 1% P/S. The pellet was then resuspended in 5 mL of red blood cell lysis buffer and centrifuged again at 1500 rpm for 5 min. Following an additional wash with 10 mL of Dhank’s buffer (1% P/S), the pellet was resuspended in an appropriate volume of Matrigel at a density of 2×10⁵ cells per 50 μL of Matrigel. The cell-Matrigel suspension was aliquoted (50 μL per well) into a 24-well plate and incubated at 37 °C in a cell culture incubator for 10 min to solidify. Subsequently, 1 mL of specialized culture medium was added to each well. The medium was replaced every 4 days to support cell growth. To determine the GEM half-maximal inhibitory concentration (IC50) of the organoids, researchers tested a series of drug concentrations and calculated the IC50 values. During the culture period, the status of the organoids was monitored daily. The presence of relatively intact organoids retained in the culture wells indicated drug tolerance, while blackened edges and shrinkage of the organoids suggested growth inhibition.

### Calcein-AM/PI double staining assay

PC cells transfected with the target gene were harvested at various time points (0, 24, 48, 72 h) post-transfection and seeded in 96-well plates at a density of 1×10⁵ cells/mL. The following day, cells were incubated with staining solution containing 1 μM Calcein-AM and 4 μM PI in culture medium at 37 °C for 15 min. Following centrifugation at 1,000 × g for 5 min, the staining solution was carefully removed. Cells were gently washed with PBS and resuspended in fresh buffer. Dual-color fluorescence imaging was performed immediately using an Olympus IX83 inverted fluorescence microscope under 490 nm excitation to simultaneously visualize live cells (green, Calcein fluorescence) and dead cells (red, PI nuclear staining).

### Cell viability assay

The Cell Counting Kit-8 (CCK-8) assay was utilized to assess cell viability. A total of 100 μl of PC cells at a concentration of 5×10^4^ cells/ml were seeded into 96-well plates and cultured for 12 h at 37 °C under 5% CO2, followed by the indicated treatments. Subsequently, the cells were incubated with CCK-8 solution (#C0037, Beyotime, Shanghai, China) for 1.5 h at 37 °C. The optical density (OD) value at 450 nm was measured using an iMark microplate reader (Bio-Rad). Data were presented as percentages after normalization to the control group.

### Glycolysis stress test

According to the manufacturer’s protocols, extracellular acidification rate (ECAR) and oxygen consumption rate (OCR) of PC cells were analyzed using the Seahorse XF24 Extracellular Flux Analyzer (Agilent Seahorse). Briefly, cells were seeded at 5 × 10^4^ cells/well in Seahorse XF-specific microplates, followed by assessment of glycolytic function with the XF Glycolysis Stress Test Kit (Agilent).

### Immunofluorescent staining

PC cells subjected to various treatments were seeded in confocal dishes. After 24-hour culture, cells were fixed with 4% paraformaldehyde for 30 min at room temperature, followed by permeabilization in PBS containing 0.3% Triton X-100 for 10 min. Following two PBS washes, nonspecific binding was blocked with 3% fetal bovine serum for 1 h. Primary antibodies were applied and incubated overnight at 4 °C. Subsequently, cells were incubated with species-matched secondary antibodies conjugated to either Alexa Fluor 594 or Alexa Fluor 488 (Invitrogen, Shanghai, China) for 1 h at room temperature. Nuclei were counterstained with DAPI (Sigma-Aldrich, St. Louis, USA). Fluorescence images were acquired using a laser scanning confocal microscope (Leica Microsystems, Germany).

### Glucose uptake and lactate production assay

For glucose uptake assays‌, cells were seeded in 6-well plates and cultured for 24 h. Upon reaching 80% confluency, sequential treatments were performed: incubation in low-glucose medium (5 mM glucose) for 4 h, replacement with glucose-free medium for 30 min, followed by treatment with glucose-free medium containing 2.5 µg/mL 2-NBDG (2-deoxyglucose analog) for 30 min. Cells were then washed three times with PBS, harvested, and analyzed for fluorescence intensity using flow cytometry. ‌Lactate production was quantified using a Lactate Colorimetric/Fluorometric Assay Kit according to the manufacturer’s protocol. Cells were seeded in 6-well plates and cultured in complete medium for 24 h. The medium was then replaced with fresh medium containing 5 mM glucose and incubated for 15 h. Subsequently, culture supernatants were collected for lactate measurement using a microplate reader, while cells were lysed for protein normalization and Western blot analysis.

### Animal experiments

This animal study was conducted in compliance with ethical guidelines approved by the Animal Care and Ethics Committee of the Second Affiliated Hospital, Zhejiang University School of Medicine (2024[260]). Male BALB/c-nu/nu nude mice (6–8 weeks old, mean weight 18 g ± 1.5 g) purchased from Charles River Laboratories (Beijing, China) were housed under controlled conditions (temperature: 22–24 °C; humidity: 40–60%; 12-h light/dark cycle). For subcutaneous xenograft modeling, mice were randomized into groups (n = 6/group) and inoculated unilaterally in the flank with 2×10⁶ pLEX-lentivirus-infected cells expressing firefly luciferase cDNA; experimental setups included: (i) GEM-resistant pancreatic cancer (GEM-R PC) cells with shNC, (ii) GEM-R PC cells with shVRK2, (iii) GEM-R PC cells with shNC + TPI1 overexpression, and (iv) GEM-R PC cells with shVRK2 + TPI1 overexpression. Tumor dimensions were measured every 5 days, with volume calculated as V = 1/2(length × width²). Once the largest tumor reached ~100 mm³, gemcitabine therapy commenced (i.p., 100 mg/kg, twice weekly for 42 days). After 6 weeks, tumor burden was assessed via in vivo imaging (IVIS) before euthanasia, followed by tumor weighing and histological evaluation. For the mithramycin A assay, mice received subcutaneous injections of 2 × 10⁶ luciferase-expressing cells from: (i) GEM-sensitive PC (GEM-S PC) cells with Vector, (ii) GEM-S PC cells with VRK2 overexpression, (iii) GEM-S PC cells with Vector + mithramycin A (i.p., 0.2 mg/kg, twice weekly), and (iv) GEM-S PC cells with VRK2 overexpression + mithramycin A (i.p., 0.2 mg/kg, twice weekly). Gemcitabine (i.p., 100 mg/kg, twice weekly for 42 days) was administered at xenograft volumes of ~100 mm³; tumor progression was monitored by IVIS until endpoint euthanasia.

### Haematoxylin & eosin (H&E) staining and immunohistochemical analysis

Formalin-fixed tissue specimens were paraffin-embedded, serially sectioned at 5μm thickness, and subjected to dewaxing in xylene followed by gradient alcohol hydration. Routine histological evaluation was performed using hematoxylin and eosin (H&E) staining. For immunohistochemical (IHC) staining, antigen retrieval was followed by blocking with goat serum for 1 h at room temperature, overnight incubation with primary antibodies at 4 °C, and 1-hour incubation with secondary antibodies at room temperature. Immunoreactivity was visualized using a hydrogen peroxide-diaminobenzidine (DAB) substrate kit, counterstained with Mayer’s hematoxylin (DAKO). After imaging under an Olympus light microscope, semi-quantitative scoring was performed by multiplying staining intensity (0: negative; 1: weak; 2: moderate; 3: strong) by the percentage of positive cells (0-100%).

### Statistical analysis

Data are expressed as mean ± standard deviation (SD) from a minimum of three independent biological replicates. All statistical analyses were conducted using GraphPad Prism 7 (GraphPad Software, USA). For two-group comparisons, two-tailed Student’s t-tests were performed. Comparisons involving three or more groups were analyzed using one-way or two-way analysis of variance (ANOVA) with Bonferroni post hoc correction. The Kaplan-Meier method was utilized to draw survival curves. Spearman’s correlation test was employed in correlation analyses. For dose–response curve analysis of PC cells, nonlinear regression was employed to calculate the half-maximal inhibitory concentration (IC50). A *P*-value < 0.05 was considered statistically significant. All in vitro experiments were repeated at least three times, and the exact number of animals per experimental group is specified in the respective figure legends.

## Supplementary information

Supplementary figure

Supplementary Materials and Methods

Original Data

## Data Availability

All datasets generated and analyzed during this study are included in this published article and its supplementary information files. Any additional data is available from the corresponding author upon reasonable request.
