# Supplementary material for: Vaccinia-related kinase 2 confers pancreatic cancer with gemcitabine resistance through TPI1-driven aerobic glycolysis
Source: Cell Death Discov. 2026 Aug 17;12:455. doi: 10.1038/s41420-026-03303-8 (PMC13754781; doi:10.1038/s41420-026-03303-8)

Figure 1

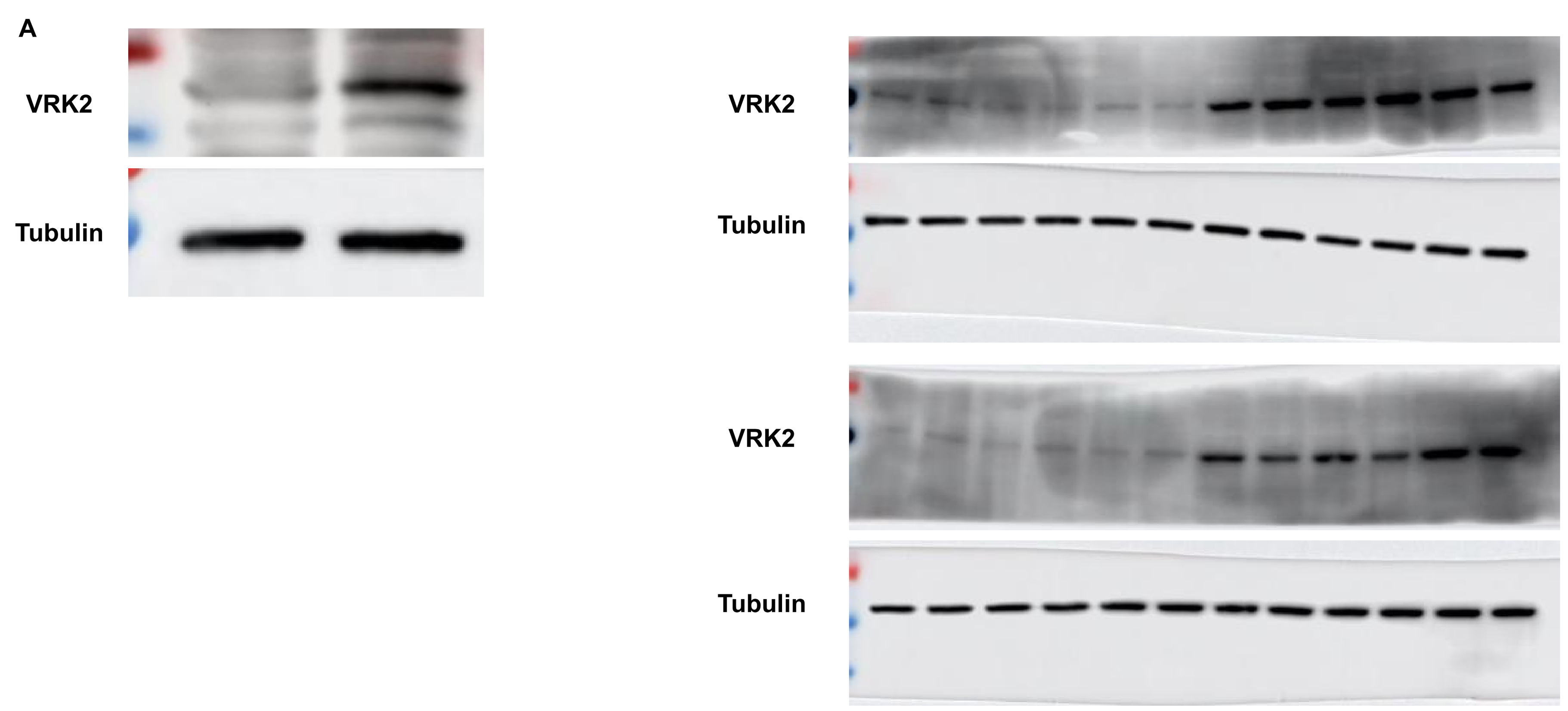

Figure 2

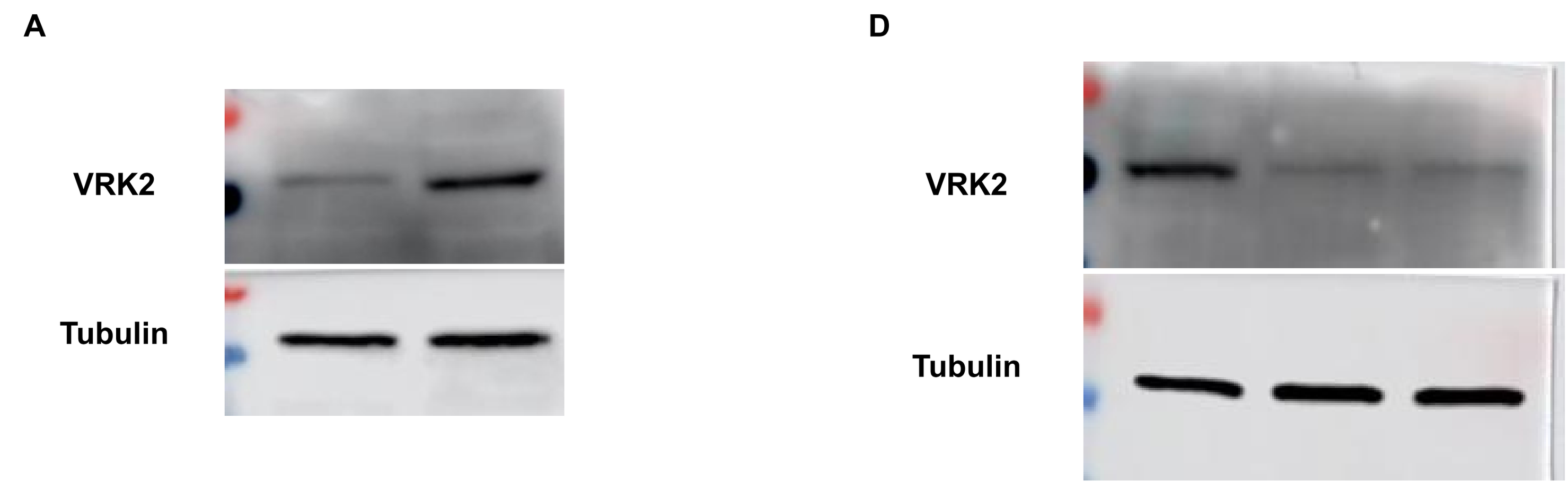

Figure 3

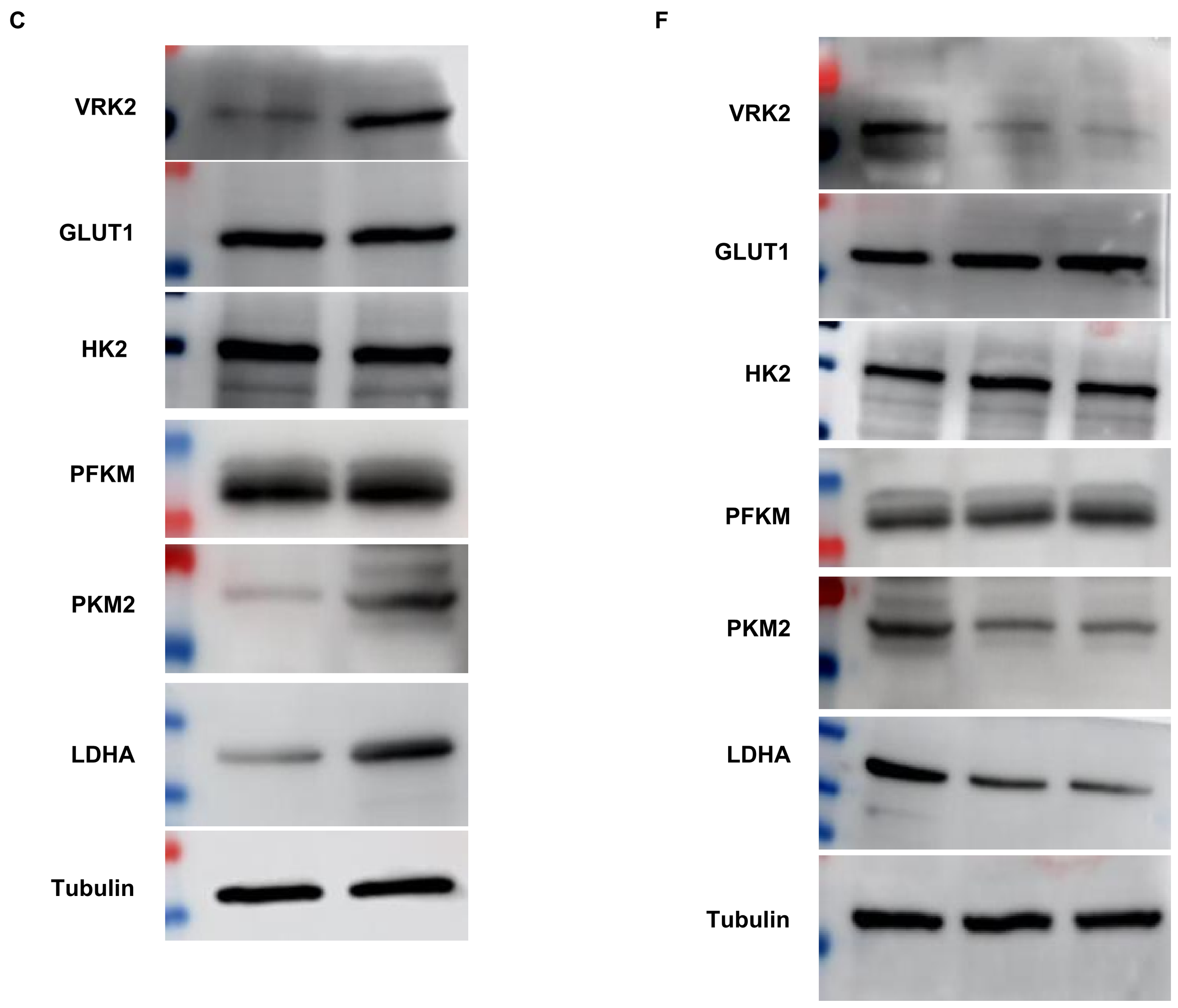

Figure 4

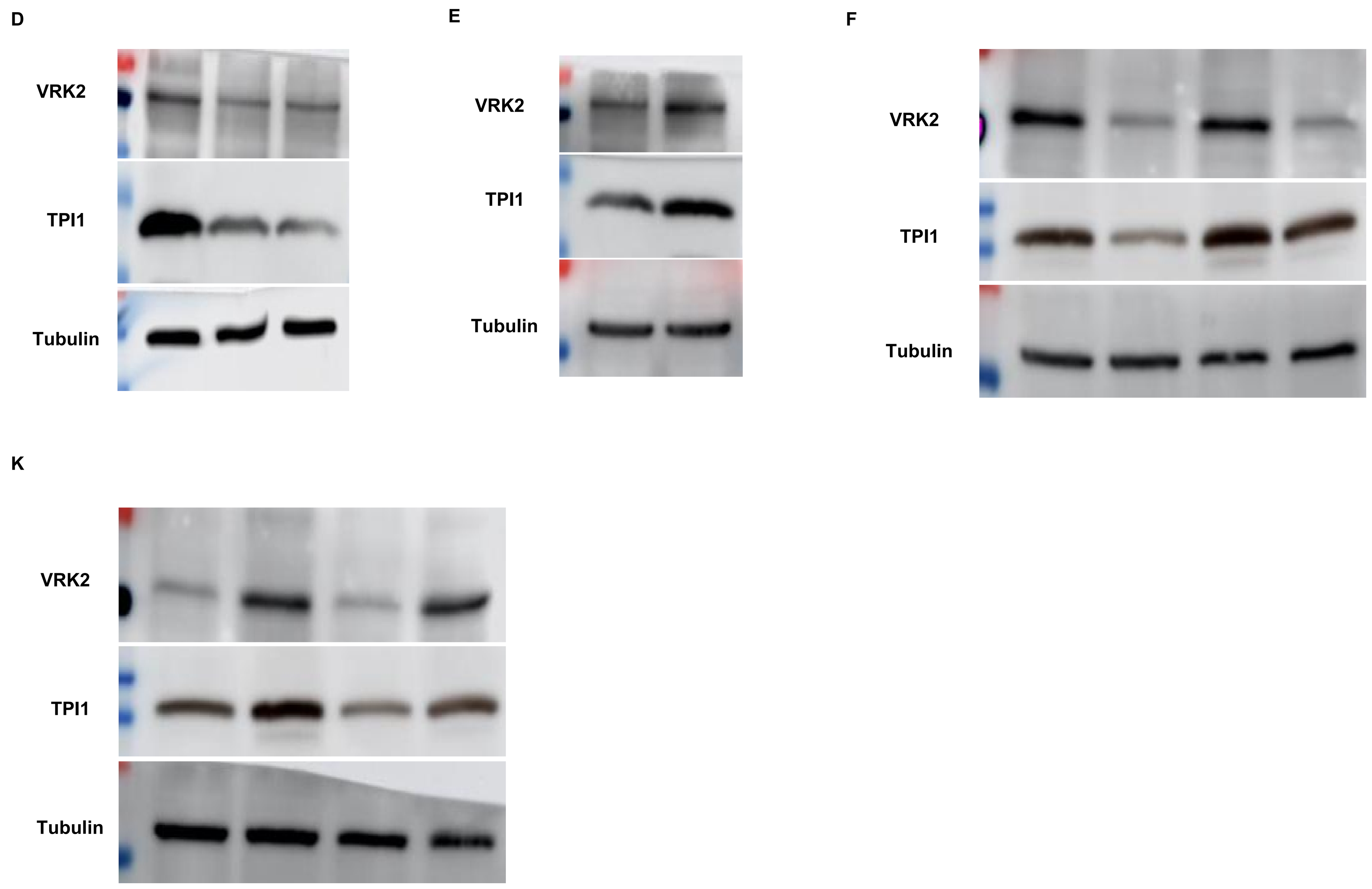

Figure 5

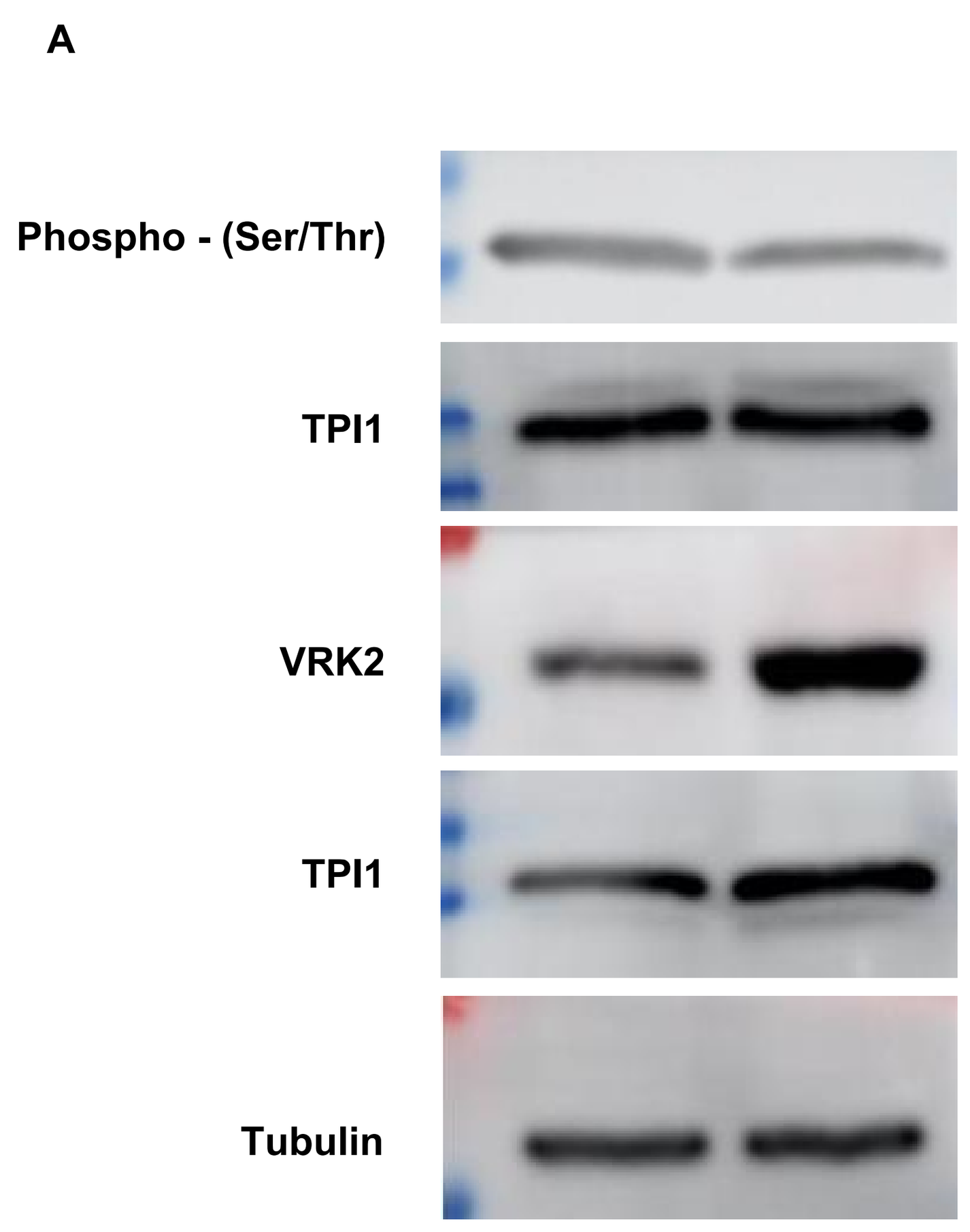

Figure 6

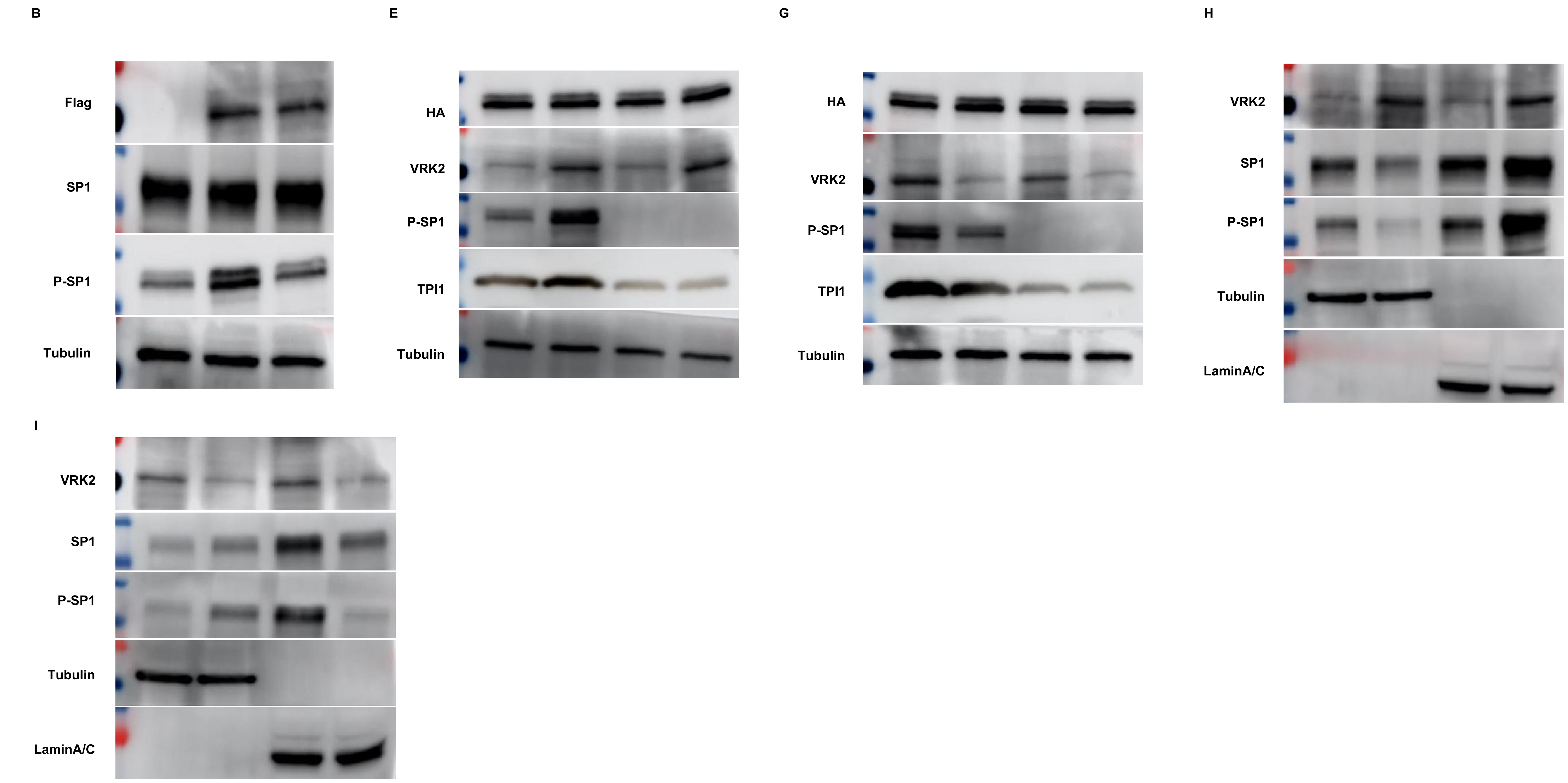

Figure 7

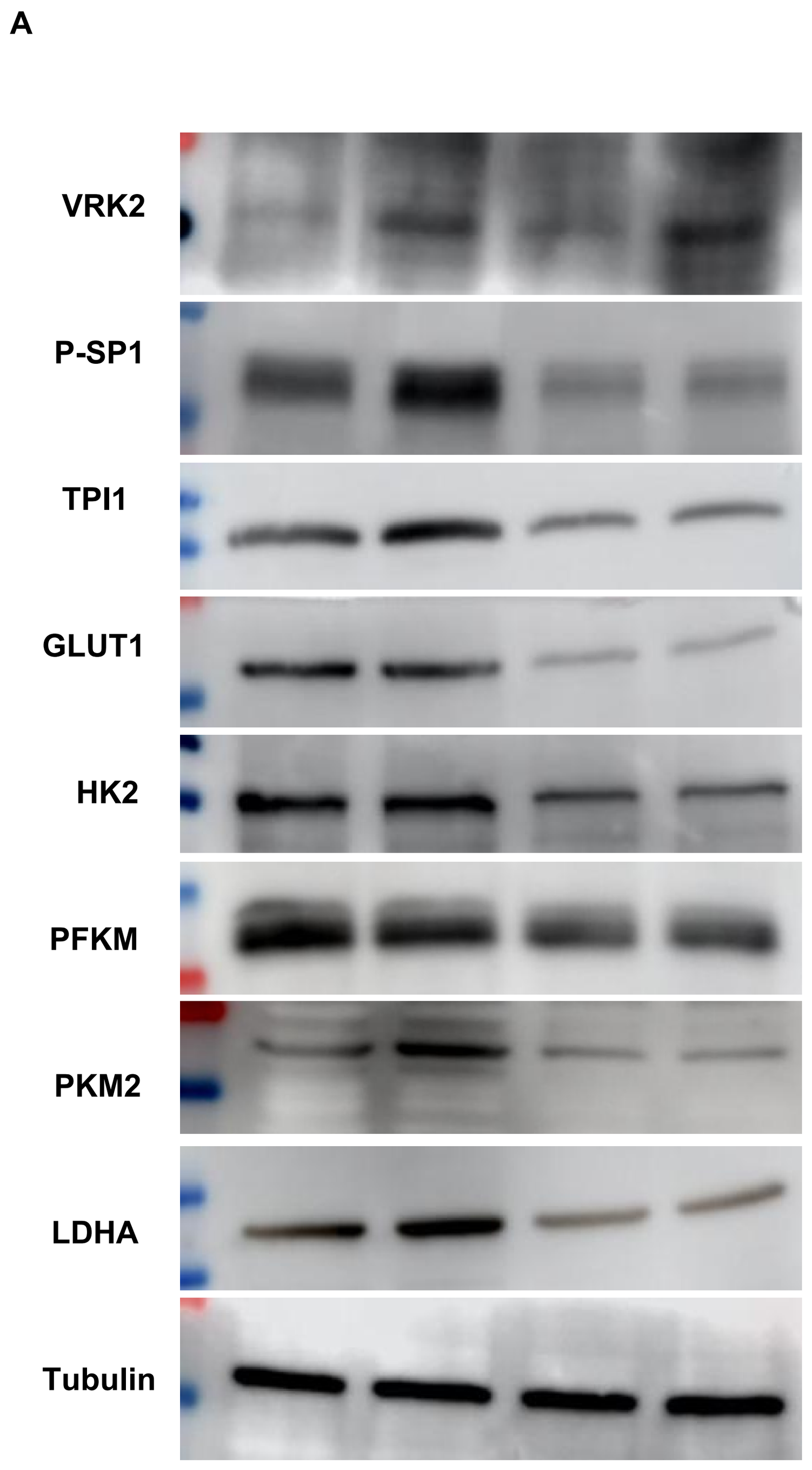
A
VRK2
P-SP1
TPI1
GLUT1
HK2
PFKM
PKM2
LDHA
Tubulin

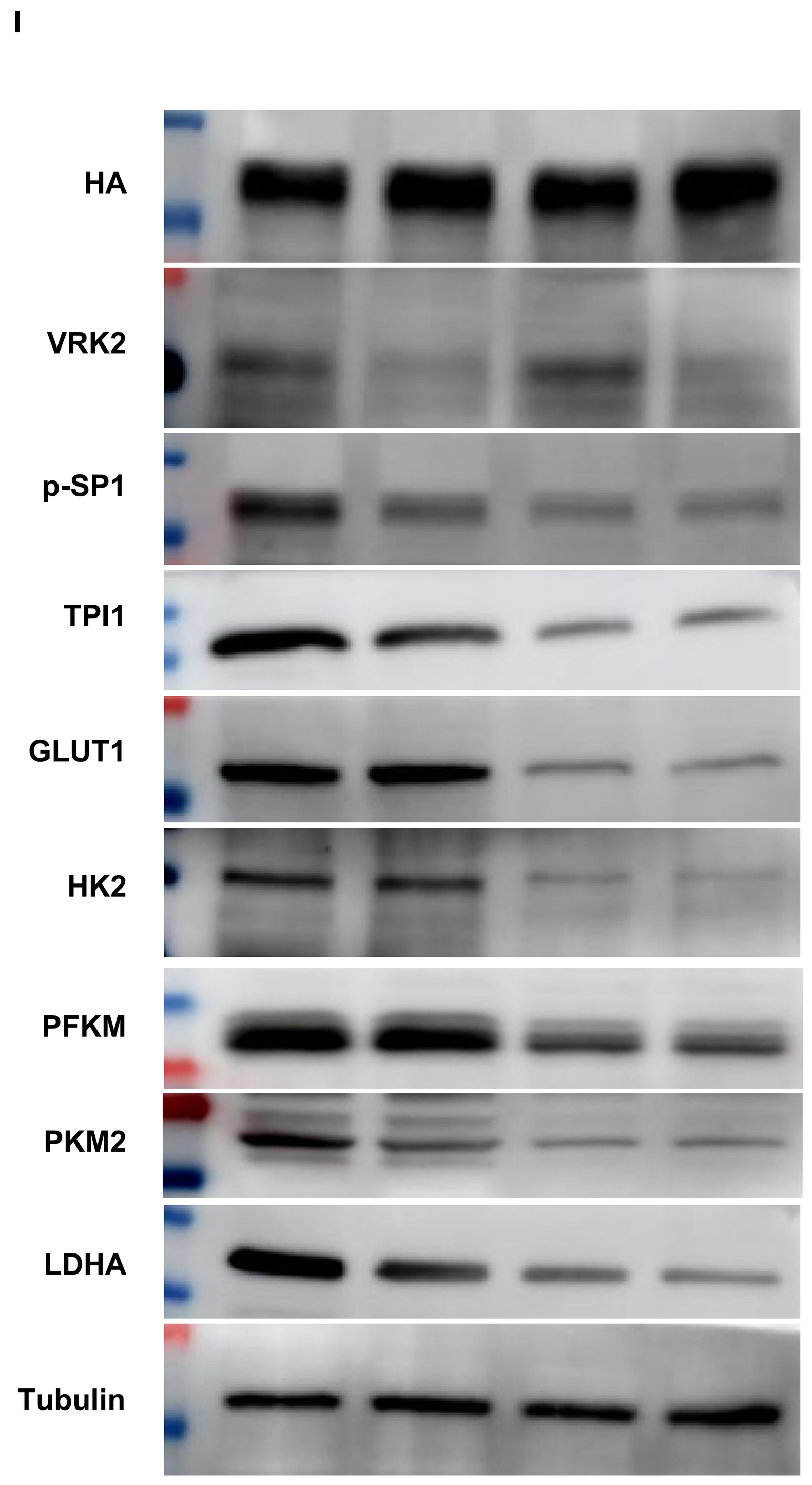
I
HA
VRK2
p-SP1
TPI1
GLUT1
HK2
PFKM
PKM2
LDHA
Tubulin

Supplementary Figure 1

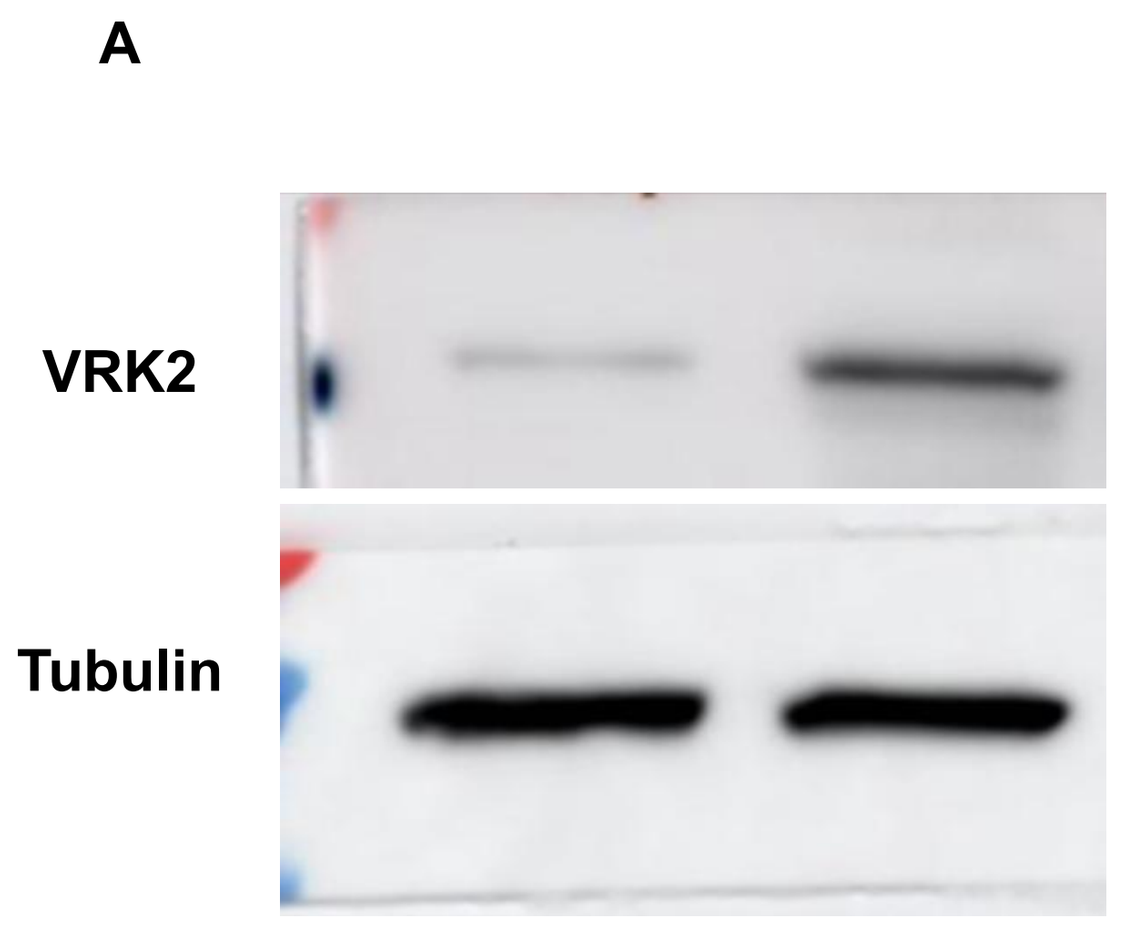

Supplementary Figure 2

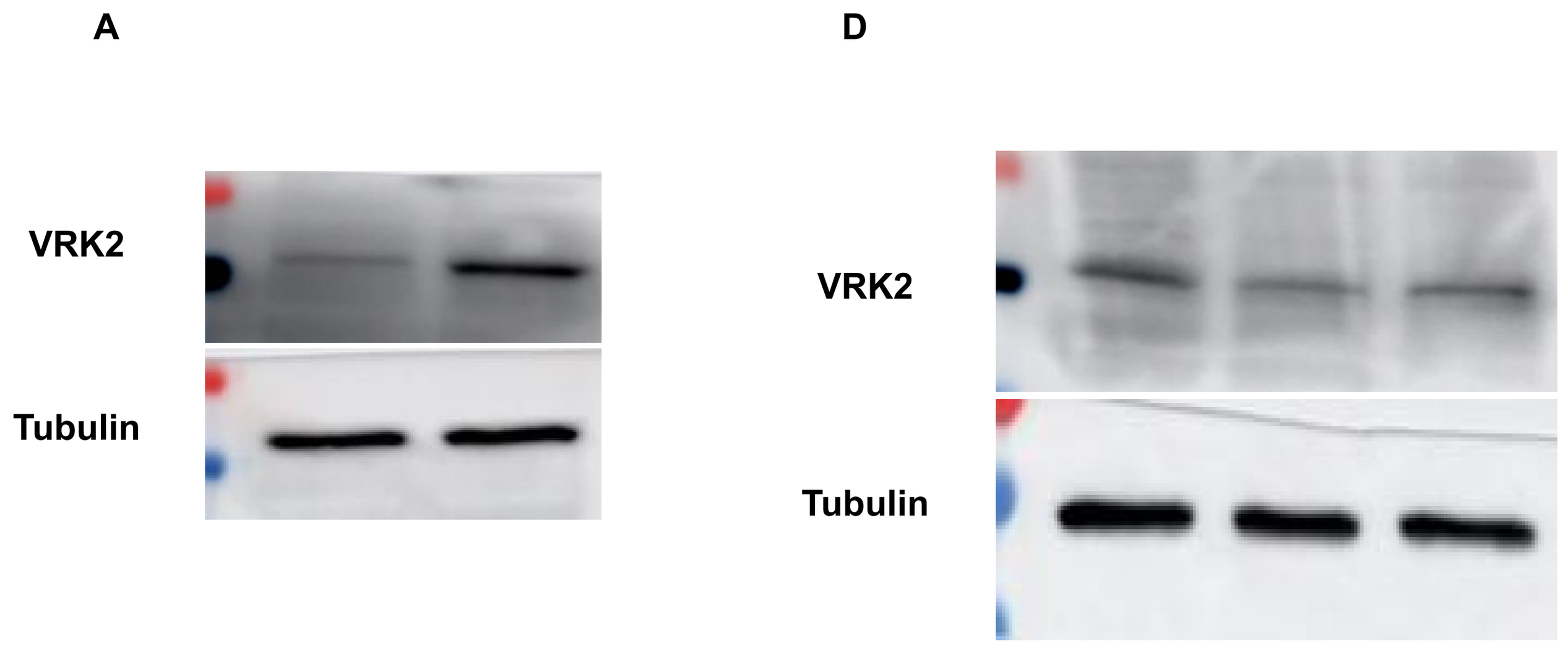

Supplementary Figure 3

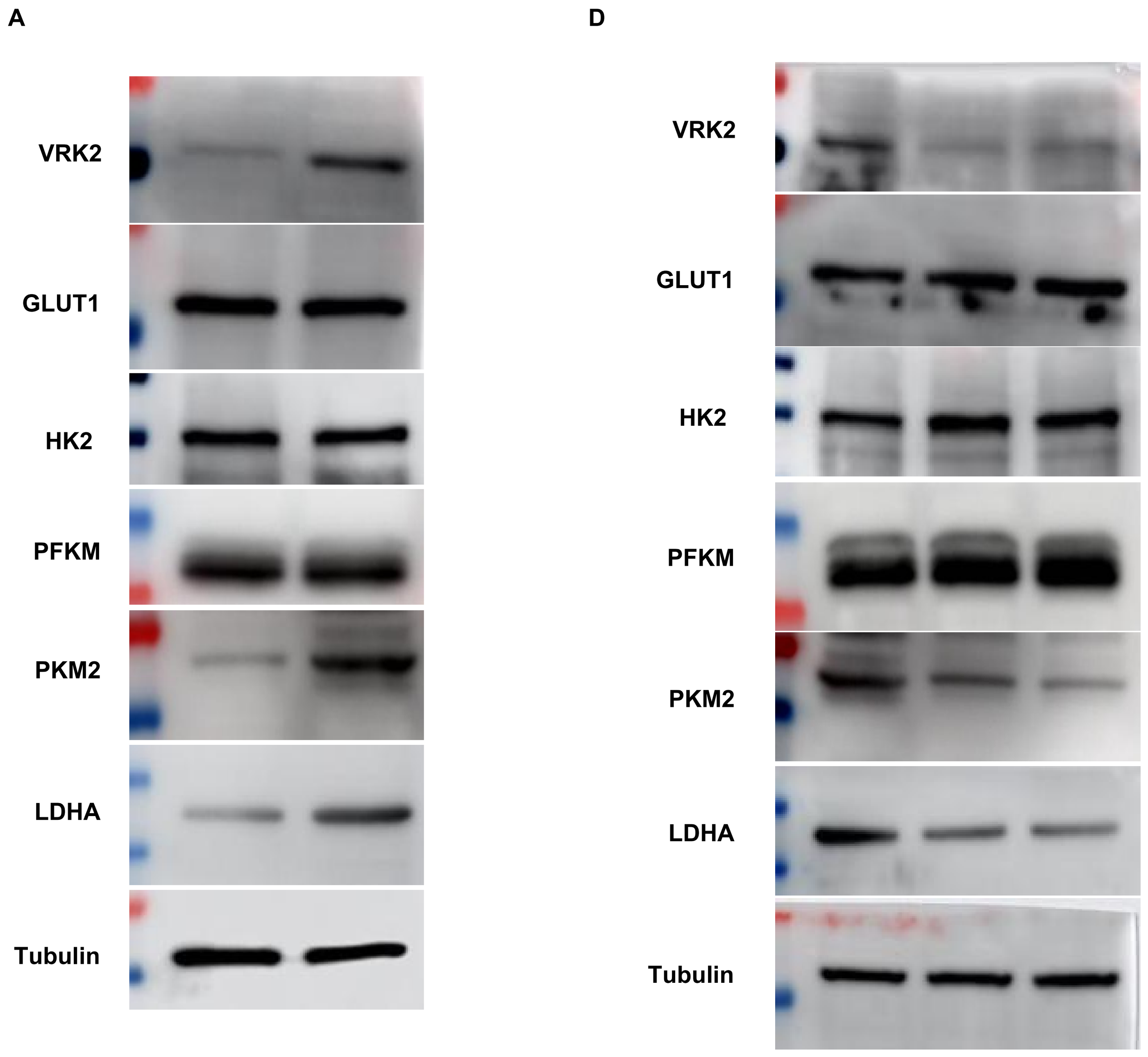

Supplementary Figure 4

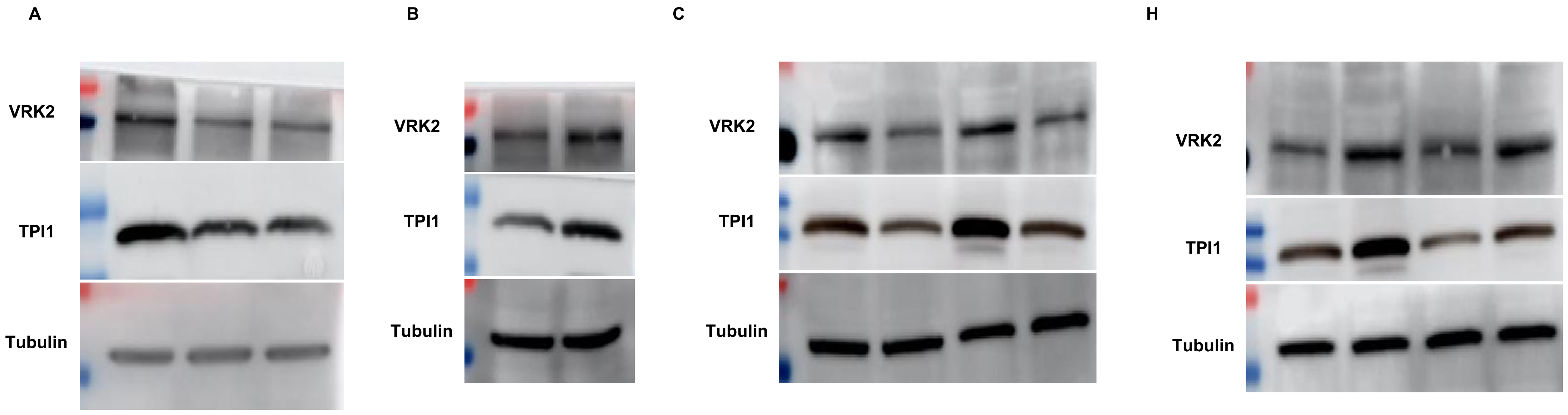

Supplementary Figure 5

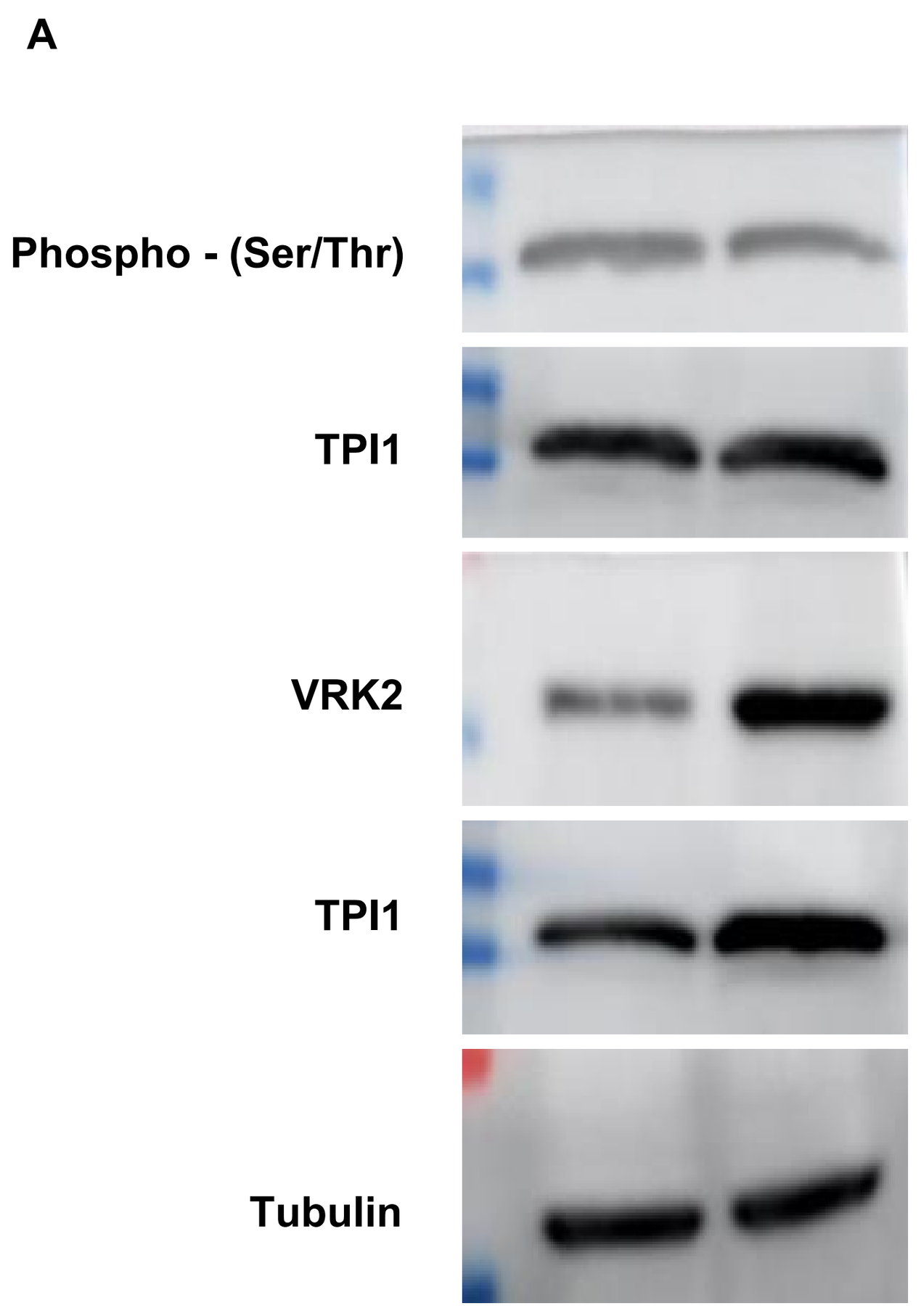

Supplementary Figure 6

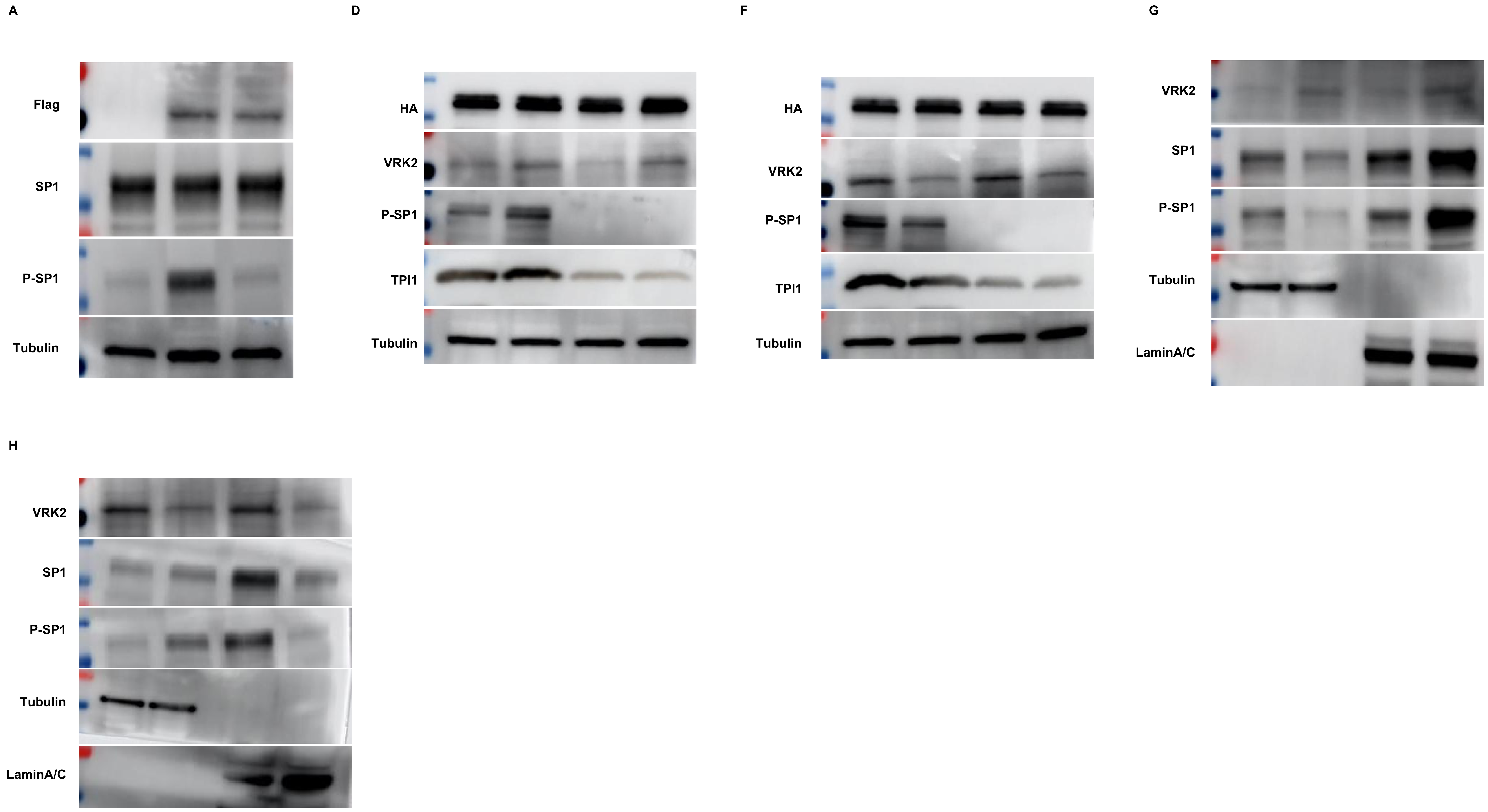

Supplement: Supplementary file 1 [file 41420_2026_3303_MOESM3_ESM.pdf]
