# Supplementary material for: Vaccinia-related kinase 2 confers pancreatic cancer with gemcitabine resistance through TPI1-driven aerobic glycolysis
Source: Cell Death Discov. 2026 Aug 17;12:455. doi: 10.1038/s41420-026-03303-8 (PMC13754781; doi:10.1038/s41420-026-03303-8)
Supplement: Supplementary file 2 [file 41420_2026_3303_MOESM1_ESM.docx]

**Supplementary Figure Legends**

**Supplementary Fig.1 Elevated expression of VRK2 in GEM-resistant PC cells**

**A, B** The IC50 values of PANC-1 and SW1990 treated with GEM determined by CCK8 assay(n=5 for GEM-sensitive cells and n=5 for GEM-resistant cells); PANC-1 GEM-R VS PANC-1 GEM-S: 7.507(5.883-9.616) VS 1.186(0.9076-1.555) p＜0.001. SW1990 GEM-R VS SW1990 GEM-S: 1.772(1.181-2.664) VS 0.4187(0.2788-0.6348) p＜0.001. **C** Immunoblot for protein expression of VRK2 in GEM-resistant SW1990 cells and GEM-sensitive SW1990 cells. **D** mRNA of VRK2 in GEM-resistant SW1990 cells and GEM-sensitive SW1990 cells tested by RT-qPCR. n = 3; **p <0.01.

**Supplementary Fig.2 VRK2 promotes the GEM resistance *in vitro* and *in vivo***

**A,B** VRK2 overexpression in GEM-sensitive SW1990 cells and VRK2 knockdown in GEM-resistant SW1990 cells upon GEM treatment (1 μM for GEM‑resistant cells; 0.3 μM for GEM‑sensitive cells) were validated by Western blotting. **C,E** Calcein-AM/PI double staining was used to evaluate SW1990 cell viability following VRK2 overexpression or knockdown in the presence of differential GEM concentrations (1 μM for GEM-resistant cells and 0.3 μM for GEM-sensitive cells). **D,F** Time-dependent cell viability of SW1990 cells was assessed via CCK-8 assay upon VRK2 overexpression or knockdown, with sustained GEM treatment at line-specific concentrations (0.3 μM for GEM-sensitive cells and 1 μM for GEM-resistant cells).**p <0.01; ***p <0.001. **G, H** The IC50 values of SW1990 cell following VRK2 overexpression or knockdown in the presence of differential GEM concentrations determined by CCK8 assay (n=5 for GEM-sensitive cells and n=5 for GEM-resistant cells); Vector VS Flag-VRK2: 1.31(0.95-1.80) VS 8.80(6.40-12.22) p＜0.001. shNC VS shVRK2-1 VS shVRK2-2: 1.49(1.14-1.96) VS 0.76(0.60-0.79) VS 0.63（0.50-0.79）p＜0.001. **I** Mice injected with luciferase-expressing vector or VRK2-overexpressing GEM-sensitive SW1990 cells were assessed by IVIS imaging system. **J** Volume of tumors in the mice with vector or VRK2-overexpressing GEM-sensitive SW1990 cells. Tumor volumes are presented as the mean ± SD, n=6; ***p < 0.001. **K** Weight of tumor in the mice with vector or VRK2-overexpressing GEM-sensitive SW1990 cells. n = 6; ***p <0.001. **L** Mice injected with luciferase-expressing shNC or shVRK2 GEM-resistant SW1990 cells were assessed by IVIS imaging system. **M** Volume of tumors in the mice with shNC or shVRK2 GEM-resistant SW1990 cells. Tumor volumes are presented as the mean ± SD, n=6; *p <0.05; ***p < 0.001. **N** Weight of tumor in the mice with shNC or shVRK2 GEM-resistant SW1990 cells. n = 6; ***p <0.001. **O** IHC analysis of VRK2, Ki67 expression of tumors in the mice with vector or VRK2-overexpressing GEM-sensitive SW1990 cells. n=6; ***p < 0.001. For 100×figures, the scale bar indicates 100 μm; For 400×figures, the scale bar indicates 50 μm. **P** IHC analysis of VRK2, Ki67 expression of tumors in the mice with shNC or shVRK2 GEM-resistant SW1990 cells. n=6; ***p < 0.001. For 100×figures, the scale bar indicates 100 μm; For 400×figures, the scale bar indicates 50 μm.

**Supplementary Fig.3 Aerobic glycolysis is involved in VRK2-mediated GEM resistance in PC**

**A** Immunoblot for protein expression of GLUT1, HK2, PFKM, PKM2 and LDHA in VRK2-overexpressing GEM-sensitive SW1990 cells treated with 0.3 μM GEM. **B, C** Relative levels of glucose uptake and lactate production in VRK2-overexpressing GEM-sensitive SW1990 cells treated with 0.3 μM GEM. n=3; **p < 0.01; ***p < 0.001. **D** Immunoblot for protein expression of GLUT1, HK2, PFKM, PKM2 and LDHA in VRK2-knockdown GEM-resistant SW1990 cells treated with 1 μM GEM. **E, F** The relative levels of glucose uptake and lactate production of GEM-resistant SW1990 cells with VRK2 knockdown in the presence of GEM (1 μM). n=3; ***p < 0.001. **G, H** The effect of VRK2 overexpression on ECAR in GEM-sensitive SW1990 cells treated with 0.3 μM GEM. n=3; ***p < 0.001. **I, J** The effect of VRK2 knockdown on ECAR in VRK2-knockdown GEM-resistant SW1990 cells treated with 1 μM GEM. n=3; ***p < 0.001. **K, L** The relative levels of glucose uptake and lactate production in VRK2-overexpressing GEM-sensitive SW1990 cells treated with 0.3 μM GEM in the presence or absence of PFK158(30 μM). n=3; *p <0.05; **p < 0.01; ***p < 0.001. **M, N** The effect of VRK2 overexpression on ECAR in GEM-sensitive SW1990 cells treated with 0.3 μM GEM in the presence or absence of PFK158(30 μM). n=3; *p <0.05; **O** Effects of VRK2 overexpression on cell viability in SW1990 cells exposed to 0.3 μM GEM with or without PFK158(30 μM). n=3;*p < 0.05; ***p < 0.001.

**Supplementary Fig.4 VRK2 promotes glycolysis and GEM-resistance via TPI1 in PC**

**A** Immunoblot for protein expression of TPI1 in VRK2-knockdown GEM-resistant SW1990 cells treated with 1 μM GEM. **B** Immunoblot for protein expression of TPI1 in VRK2-overexpressing GEM-sensitive SW1990 cells treated with 0.3 μM GEM. **C** The expression of TPI1 in VRK2-knockdown GEM-resistant SW1990 cells in the presence of GEM (1 μM) without or with TPI1 overexpression. **D, E** The relative levels of glucose uptake and lactate production in VRK2-knockdown GEM-resistant SW1990 cells in the presence of GEM (1 μM) without or with TPI1 overexpression. n=3; *p < 0.05; **p < 0.01; ***p < 0.001. **F, G** ECAR in VRK2-knockdown GEM-resistant SW1990 cells in the presence of GEM (1 μM) without or with TPI1 overexpression. n=3; ***p < 0.001. **H** The expression of TPI1 in VRK2-overexpressing GEM-sensitive SW1990 cells in the presence of GEM (0.3 μM) without or with TPI1 knockdown. **I, J** The relative levels of glucose uptake and lactate production in VRK2-overexpressing GEM-sensitive SW1990 cells in the presence of GEM (0.3 μM) without or with TPI1 knockdown. n=3; **p < 0.01; ***p < 0.001. **K, L** ECAR in VRK2-overexpressing GEM-sensitive SW1990 cells in the presence of GEM (0.3 μM) without or with TPI1 knockdown. n=3; ***p < 0.001. **M** Mechanistic diagram illustrating isotopic tracing using positionally labeled [1,2-^13^C_2_] glucose, where hexagons represent carbon atoms in each metabolite. Purple hexagons denote labeled carbons and their direct contribution to lipid synthesis via DHAP. Blue hexagons demonstrate TPI1-mediated conversion of DHAP to GAP, along with downstream glycolytic intermediates and alternative flux through the oxidative pentose phosphate pathway. **N, O** Carbons (M+2) enrichment in GAP and 3-PG in VRK2-knockdown GEM-resistant SW1990 cells in the presence of GEM (1 μM) without or with TPI1 overexpression. n=3; **p < 0.01; ***p < 0.001. **P, Q** Carbons (M+2) enrichment in GAP and 3-PG in VRK2-overexpressing GEM-sensitive SW1990 cells in the presence of GEM (0.3 μM) without or with TPI1 knockdown. n=3; **p < 0.01; ***p < 0.001. **R, S** The viability of 1 μM GEM-exposed SW1990 cells following VRK2 knockdown with or without TPI1 overexpression was evaluated by Calcein-AM/PI double staining and CCK-8 assay. n=3; ***p < 0.001. **T, U** The viability of 0.3 μM GEM-exposed SW1990 cells following VRK2 overexpression without or with TPI1 knockdown was tested by calcein-AM/PI double staining and CCK8 assay. n=3; ***p < 0.001. **V** Following GEM treatment, in vivo bioluminescence was detected using an IVIS imaging system in mice inoculated with luciferase-expressing, GEM-sensitive PANC-1 cells with vector/VRK2 overexpression and with or without TPI1 knockdown. **W** The tumor volume of mice implanted with GEM-sensitive PANC-1 cells with distinct genetic modifications was evaluated upon GEM intervention. Tumor volumes are presented as the mean ± SD, n=6; ***p < 0.001.

**Supplementary Fig.5 SP1 is identified as a transcriptional factor regulating the expression of TPI1**

**A** Immunoblot for protein expression of p-TPI1 and TPI1 in VRK2-overexpressing GEM-sensitive SW1990 cells treated with 0.3 μM GEM. **B** The relative mRNA level of TPI1 in VRK2-overexpressing GEM-sensitive SW1990 cells treated with 0.3 μM GEM. n=3; ***p < 0.001. **C** Positive correlations‌ between TPI1 and 12 potential transcription factors validated by GEPIA analysis. **D** Relative mRNA levels of TPI1 in SW1990 cells with potential transcription factors overexpression. n=3; NS, no significant; ***p < 0.001. **E** Relative transcriptional activity of TPI1 in SW1990 cells with potential transcription factors overexpression. n=3; NS, no significant; ***p < 0.001. **F** The relative transcriptional activity of TPI1 in SW1990 cells co-transfected with an SP1 expression plasmid and truncated constructs containing SP1-binding regions within the TPI1 promoter. n=3; NS, no significant; ***p < 0.001. **G** Relative luciferase activities of different reporters containing wild-type (WT) and mutated (MUT) sequences of the TPI1 promotor in SW1990 cells expressing SP1 plasmid. Filled circles represent the putative SP1-binding sites, whereas ‘**×**’ marks the mutated SP1-binding sites. n=3; NS, no significant; ***p < 0.001. **H** The relative transcriptional activity of TPI1 in SW1990 cells co-transfected with PDGF-BB expression plasmid and truncated constructs containing SP1-binding regions within the TPI1 promoter. n=3; NS, no significant; ***p < 0.001. **I** Relative luciferase activities of different reporters containing wild-type (WT) and mutated (MUT) sequences of the TPI1 promotor in SW1990 cells expressing PDGF-BB plasmid. Filled circles represent the putative SP1-binding sites, whereas ‘×’ marks the mutated SP1-binding sites. n=3; NS, no significant; ***p < 0.001.

**Supplementary Fig.6 VRK2 phosphorylates SP1 at Ser2 and promotes its nuclear import**

**A** Western blot analysis of p-SP1 and total SP1 expression in SW1990 cells harbouring wild-type VRK2 or kinase-dead VRK2 (K61A) following 0.3 μM GEM stimulation. **B** The effect of VRK2 on TPI1 transcriptional activity in SP1-knockout SW1990 cells reconstituted with wild-type SP1 or SP1 (S2A) mutant plasmids under 0.3 μM GEM treatment. n=3; NS, no significant; ***p < 0.001. **C, D** The effect of VRK2 on TPI1 mRNA and protein levels in SP1-knockout SW1990 cells reconstituted with wild-type SP1 or SP1 (S2A) mutant plasmids upon 0.3 μM GEM treatment. n=3; NS, no significant; ***p < 0.001. **E, F** TPI1 mRNA and protein levels regulated by VRK2 knockdown in SP1-knockout SW1990 cells reconstituted with wild-type SP1 or SP1 (S2A) mutant plasmids following 1 μM GEM treatment. n=3; NS, no significant; ***p < 0.001. **G, H** Cytoplasmic and nuclear protein levels of SP1 and p-SP1 in VRK2-overexpressing or VRK2-knockdown SW1990 cells under prolonged GEM treatment at cell line-specific concentrations (0.3 μM for GEM-sensitive cells and 1 μM for GEM-resistant cells). **I** Modulation of SP1 subcellular localization by VRK2 in SP1-deficient SW1990 cells reconstituted with wild-type SP1 or S2A-mutant SP1 under 0.3 μM GEM exposure. Scale bar indicates 20 μm.

**Supplementary Fig.7 Serum indicators of liver and kidney function among the different groups of mice during treatment**

**A-D** Quantitative analysis of indicated biochemistry indices for liver and kidney function in mice inoculated with GEM-sensitive PANC-1 cells transfected with vector, VRK2-overexpressing construct, vector + mithramycin A, or VRK2-overexpressing construct + mithramycin A, followed by GEM treatment. n=6; NS, no significant.
