# Supplementary material for: Vaccinia-related kinase 2 confers pancreatic cancer with gemcitabine resistance through TPI1-driven aerobic glycolysis
Source: Cell Death Discov. 2026 Aug 17;12:455. doi: 10.1038/s41420-026-03303-8 (PMC13754781; doi:10.1038/s41420-026-03303-8)
Supplement: Supplementary file 3 [file 41420_2026_3303_MOESM2_ESM.docx]

**Cell transfection**

Lentiviral vectors encoding short hairpin RNAs (shRNAs) targeting VRK2 (shVRK2), TPI1 (shTPI1), SP1(shSP1) and the corresponding negative control shRNA (shNC) were obtained from Genechem (Shanghai, China). ‌Cells transduced with these vectors‌ were selected using puromycin for 4 weeks. Plasmid constructs for overexpressing VRK2, TPI1, wild-type (WT) SP1, or mutant (Mut) SP1 were transfected into PC cells using Lipofectamine 3000. Expression levels were assessed by RT-qPCR and Western blotting.

**CCK-8-based cytotoxicity assay**

GEM sensitivity of GEM-sensitive and resistant PANC-1/SW1990 cells was assessed via CCK-8. Logarithmic-phase cells were seeded into 96-well plates at 5×10³ cells/well, adhered overnight (37 °C, 5% CO₂), and then treated with serially diluted GEM. Drug-free medium and blank wells served as controls and background correction, respectively. After designated incubation, CCK-8 reagent was added at a preset volume ratio, followed by further incubation under standard conditions. Absorbance at 450 nm was measured using a microplate reader. Relative cell viability was calculated by normalizing drug-treated absorbance to untreated controls. Dose–response curves were generated via nonlinear regression, and half-maximal inhibitory concentration (IC₅₀) values were computed. All experiments included three biological replicates with triple technical repeats for statistical reliability.

**Analyses of human clinical biospecimens**

This study enrolled 24 pre-neoadjuvant chemotherapy tumor specimens from pancreatic cancer patients who met the following criteria: (1) histopathologically confirmed pancreatic adenocarcinoma; (2) exclusive treatment with gemcitabine-based neoadjuvant chemotherapy (without concurrent anticancer therapies); (3) bimonthly therapeutic response assessment via computed tomography (CT), with patients stratified into chemosensitive (complete response [CR] or partial response [PR]) and chemoresistant (stable disease [SD] or progressive disease [PD]) groups according to modified RECIST criteria. The study protocol was approved by the Institutional Review Board of The Second Affiliated Hospital, Zhejiang University School of Medicine(2024[1360]), with written informed consent obtained from all participants. Fresh specimens were promptly allocated for organoid culture, while residual tissues were flash-frozen in liquid nitrogen and archived at -80℃ to ensure biospecimen integrity.

**Quantitative real-time PCR (q-PCR)**

Total RNA was extracted from samples with indicated treatments using Trizol reagent (Invitrogen, USA), followed by reverse transcription into complementary DNA (cDNA) with the PrimeScript RT Reagent Kit (TaKaRa Bio, Japan).‌ Quantitative real-time PCR (qPCR) was then performed using SYBR Premix Ex Taq (TaKaRa Bio, Japan), according to the manufacturer’s instructions. The relative expression of target genes was calculated ‌using the 2^-△△Ct^ method, ‌with data presented as fold-change relative to the control group‌. The following primers were used for q-PCR:

Human VRK2 Forward:5’-GTGGATAGAACGCAAACAACTTG-3’;

Human VRK2 Reverse: 5’-CGGATACCTAATTGCAGGACAGT-3’;

Human TPI1 Forward: 5’-CCACTTCGCGGCGCTCTAT-3’；

Human TPI1 Reverse: 5’-CCAGTTTCCCCCAACGAAGA-3’；

Human SP1 Forward:5’-TGCCTTTTCACAGGCTCGAA-3’;

Human SP1 Reverse: 5’-TTGTGTGGCTGTGAGGTCAA-3’；

Human EGR1 Forward: 5’-CTAGTGAGCATGACCAACCCAC-3’;

Human EGR1 Reverse: 5’-CGCTGAGTAAATGGGACTGCTGT-3’；

Human MAZ Forward: 5’-GGATCACCTCAACAGTCACGTC-3’;

Human MAZ Reverse: 5’-GGCACTTTCTCCTCGTGTCGTA-3’；

Human PBX3 Forward: 5’-CAAGTCGGAGCCAATGTG-3’;

Human PBX3 Reverse: 5’-ATGTAGCTCAGGGAAAAGTG-3’；

Human CTCF Forward: 5’-GTGTTCCATGTGCGATTACG-3’;

Human CTCF Reverse: 5’-TCATGTGCCTTTTCAGCTTG-3’；

Human TCF3 Forward: 5’-CCAGACCAAACTGCTCATCCTG-3’；

Human TCF3 Reverse: 5’-TCGCCGTTTCAAACAGGCTGCT-3’；

Human ELF1 Forward: 5’-TGTCCAACAGAACGACCTAGT-3’;

Human ELF1 Reverse: 5’-GGCAGGAAAAATAGCTGGATCAC-3’；

Human E2F6 Forward: 5’-GACCTCGTTTTGATGTATCGCTG-3’；

Human E2F6 Reverse: 5’-ATACACTCTCCGCTTTCGGAC-3’；

Human GABPA Forward: 5’-GGAGGAAGTGGAGGGACTGA-3’;

Human GABPA Reverse: 5’-GCTTACACATTCAGCTGGCG-3’；

Human JUND Forward: 5’-GCGAACCAAGGATTACGGAA-3’；

Human JUND Reverse: 5’-TCTTTTTGTTTGGTTTTGTTTTGC-3’；

Human EBF1 Forward: 5’-ATGTTTGGGATTCAGGAAAGC-3’;

Human EBF1 Reverse: 5’-GTAGTGAATTCCGTTATTGGTC-3’；

Human MYC Forward: 5’-GCTGCTTAGACGCTGGATTT-3’；

Human MYC Reverse: 5’-AGCAGCTCGAATTTCTTCCA-3’；

Human β-actin Forward: 5’-TGACGTGGACATCCGCAAAG-3’；

Human β-actin Reverse: 5’- CTGGAAGGTGGACAGCGAGG-3’；

**Western blot and Co-immunoprecipitation (Co-IP)**

Cytoplasmic and nuclear proteins were extracted using the Minute™ Cytoplasmic and Nuclear Protein Extraction Kit (SC-003, Invent Biotechnologies) according to the manufacturer's protocol. Protein concentrations in the samples were quantified using the BCA assay prior to Western blot analysis. Equal amounts of protein were separated by SDS-PAGE and subsequently transferred onto polyvinylidene difluoride (PVDF) membranes. Membranes were blocked with 5% non-fat milk and then incubated sequentially with primary and secondary antibodies. For Co-IP experiments, cells were washed twice with ice-cold PBS and lysed in ice-cold lysis buffer (40 mM HEPES, 120 mM NaCl, 10 mM sodium pyrophosphate, 10 mM β-glycerophosphate, 50 mM NaF, 1 mM EDTA, 0.5 mM sodium orthovanadate, protease inhibitors) for 30 minutes on ice. Lysates were centrifuged and an aliquot of the supernatant was saved as the input control. The remaining supernatant was incubated with the primary antibody and Protein A/G agarose beads overnight at 4°C with rotation. Immunoprecipitated complexes were washed three times with wash buffer (50 mM HEPES, 150 mM NaCl, protease and phosphatase inhibitors), boiled in sample buffer, and subjected to subsequent electrophoresis and Western blot analysis.

**CRISPR-Cas9 knockout screen**

To generate SP1-knockout PC cells, we first designed single-guide RNAs (sgRNAs) using the Red Cotton™ online CRISPR design platform (Guangzhou, China; https://en.rc-crispr.com/). To design the specific target sequences for sgRNA synthesis, the primers were: SP1#1 forward (5' to 3') :CATGGATGAAATGACAGCTGTGG; SP1#1 reverse (5' to 3') TAAGTGATAATCATAGAGTGGGG; SP1#2 forward (5' to 3') GTATGTGACCAATGTACCAGTGG; SP1#2 reverse (5' to 3') CTGAAGTACCCAATGCACTGAGG. The corresponding oligonucleotide pairs were annealed and cloned into the YKO-RP003 vector (Ubigene Biotech Co., Ltd., Guangzhou). The constructed plasmids containing target-specific sgRNA sequences were then transfected into PC cells using Lipofectamine 3000 reagent. Forty-eight hours post-transfection, cells were subjected to puromycin selection (1 mg/mL) for 72 hours. Surviving cells were single-cell sorted into 96-well plates and cultured for 2-4 weeks to establish monoclonal populations. Finally, successful SP1 knockout clones were validated through PCR amplification of target loci followed by Sanger sequencing confirmation of the genetic modifications.

**Luciferase reporter gene**

Amplification and cloning of serial truncations within the TPI1 promoter region (-4000 to +1) were performed, followed by insertion into the pGL2-basic luciferase reporter vector. These reporter constructs were co-transfected into PC cells with the pBoBi-SP1 expression plasmid and the Renilla luciferase internal control plasmid (pRL-series). After 24 hours of transfection, dual-luciferase activities were measured using the Dual-Luciferase® Reporter Assay System (Promega). Firefly luciferase activity was normalized to Renilla luciferase activity for each sample.

**Metabolic Flux Analysis**

Twenty-four hours before tracer labeling, cells were harvested, counted, and seeded at 2 × 10⁶ cells per 10 cm dish in RPMI 1640 medium containing 11.1 mM glucose and 10% fetal bovine serum (FBS). One hour before the addition of isotopic tracer, the culture medium was replaced to adjust the glucose concentration to 0.5 mM, and cells were washed twice with phosphate-buffered saline (PBS) prior to incubation in custom labeling medium. The labeling medium consisted of glucose-free RPMI 1640 supplemented with 0.5 mM [1,2-¹³C₂]glucose and 10% dialyzed FBS, and the start of tracer incubation was defined as time zero (t=0). Intracellular metabolites were extracted at 1, 2, and 5 minutes after tracer addition (N=3 biological replicates per group at each time point). PBS washes were omitted during metabolite extraction to maintain precise temporal resolution.

**Targeted LC-MS/MS for hydroxylamine trapping**

The metabolite extract was separated via hydrophilic interaction chromatography (HILIC) using a Phenomenex Gemini C18 reversed-phase analytical column (50 mm × 4.6 mm, 5 μm particle size) coupled with a guard column (C18, 3.5 mm, 2 mm × 20 mm). Mobile phase A consisted of 100% aqueous solution containing 10 mM tributylamine (pH adjusted to 4.95 with 15 mM acetic acid), and mobile phase B was 100% methanol. A multi-step gradient elution program was implemented: 0-5 min, 95% A; 5-15 min, 95-90% A; 15-22 min, 90-85% A; 22-26 min, 10% A (held for 4 min); 30-33 min, 95% A (held for 7 min). The flow rate was maintained at 0.2 mL/min during 0-15 min and 30-40 min intervals, and increased to 0.3 mL/min from 15-30 min. Targeted metabolites were analyzed using an Agilent 6460 triple quadrupole LC-MS/MS system equipped with the following parameters: capillary voltage at 4.0 kV, drying gas temperature 350°C, drying gas flow rate 10 L/min, and nebulizer pressure 45 psi. Relative metabolite abundance was quantified by integrating peak areas of specific multiple reaction monitoring (MRM) transitions. Data represent three independent biological experiments, each performed with three technical replicates per condition.

**RNA-Seq and bioinformatics analysis**

Total RNA was extracted from PC cells of the shNC and shVRK2 groups using TRIzol reagent (Tiangen Biotech, Beijing). Transcriptome sequencing and bioinformatics analysis were performed by GeneChem Co., Ltd. (Shanghai). Library sequencing was conducted on the Illumina HiSeq 2000 platform with a 150-bp paired-end strategy. Raw image data were converted to sequence data through base calling using the Illumina CASAVA pipeline (version 1.8.2). Differential expression gene (DEG) analysis between the two groups (each containing two biological replicates) was performed using DESeq2 (version 1.16.1, R/Bioconductor package). DESeq2 leverages information sharing across genes to generate more accurate dispersion estimates based on mean expression levels. The Benjamini-Hochberg (BH) method was applied to adjust p-values and control the false discovery rate (FDR). Genes meeting the threshold of adjusted p-value < 0.05 were identified as DEGs, with both adjusted p-value and |log2FC| serving as significance criteria.

**Tandem Mass Tags-Mass Spectrometry analyses**

Samples were homogenized in lysis buffer (4% SDS, 1 mM dithiothreitol, 150 mM Tris-HCl pH 8.0, protease inhibitor), followed by peptide labeling wherein 10μL of TMT reagent dissolved in 40 μL acetonitrile was mixed with 100 μg peptides in 100 μL of 50 mM HEPES; after 1-hour incubation, the reaction was quenched with 8 μL of 5% (w/v) hydroxylamine. The labeled peptide mixture underwent basic-pH reversed-phase high-performance liquid chromatography fractionation in buffer A (5% acetonitrile, 10 mM ammonium bicarbonate), and was subsequently loaded onto an Agilent 300 Extend C18 column for separation using an in-line degasser and photodiode array detector (Agilent 1100 c). Resultant fractions were pooled into 24 samples, acidified with 10% formic acid/5% acetonitrile, vacuum-concentrated, and subjected to liquid chromatography-tandem mass spectrometry (LC-MS/MS) analysis on a Fusion Orbitrap mass spectrometer coupled with an Easy nLC system (Thermo Fisher Scientific), with MS/MS spectra searched against the UniProt human and decoy databases using the MASCOT search engine (v2.2, Matrix Science, UK) integrated in Proteome Discoverer 1.4 software (Thermo Electron, CA).

**MALDI-MS and LC–ESI-MS/MS analysis of phosphorylated SP1 peptide**

Kinase-treated peptides‌ were analyzed using either MALDI-MS on a Shimadzu MALDI-TOF MS 8020 instrument or subjected to in-solution digestion via filter-aided sample preparation (FASP) with 5 kDa molecular weight cutoff filters (Amicon, Millipore Sigma) using recombinant Lys-C (Promega, 1:50 enzyme-to-protein ratio). The resulting peptides were desalted and concentrated using Oasis HLB 1 cc 10 mg solid-phase extraction cartridges (Waters), eluted with 70% acetonitrile containing 0.1% trifluoroacetic acid (TFA), lyophilized to complete dryness, and reconstituted in 0.1% formic acid. Samples were then analyzed by ‌high-resolution nanoflow LC-MS/MS‌ on an Orbitrap Fusion Tribrid mass spectrometer (Thermo Scientific) equipped with an EasyNano LC system in positive-ion mode. The mobile phases consisted of: ‌Solution A‌ (0.1% formic acid in water) and ‌Solution B‌ (0.1% formic acid in 98% acetonitrile). A 5 μL aliquot of each sample was loaded onto an Acclaim PepMap 100 C18 trapping column (3 μm, 75 μm × 2 cm; Thermo Fisher Scientific) at 5 μL/min using Solution A, followed by separation on an analytical EASY-Spray column (25 cm × 75 μm ID, PepMap RSLC C18 2 μm; Thermo) at 500 nL/min. Chromatographic separation employed a gradient program: 3% to 25% Solution B over 40 min, then increased to 90% B from 40 to 45 min, with the column temperature maintained at 45°C throughout the 45-min run. Mass spectrometric acquisition utilized a ‌neutral loss-triggered fragmentation‌ strategy with multistage activation for phosphopeptide analysis over a 45-min duration. Data were processed using PEAKS X+ software with the following criteria: peptide-spectrum match FDR ≤ 2.0%, precursor mass tolerance 10.0 ppm, and fragment ion mass tolerance 0.05 Da. Phosphorylation sites were validated using an Ascore threshold of 10-20.
